# Evidence for maintenance of sex determinants but not of sexual stages in red yeasts, a group of early diverged basidiomycetes

**DOI:** 10.1186/1471-2148-11-249

**Published:** 2011-08-31

**Authors:** Marco A Coelho, Paula Gonçalves, José P Sampaio

**Affiliations:** 1 Centro de Recursos Microbiológicos (CREM), Departamento de Ciências da Vida, Faculdade de Ciências e Tecnologia, Universidade Nova de Lisboa, 2829-516 Caparica, Portugal

## Abstract

**Background:**

The red yeasts are an early diverged group of basidiomycetes comprising sexual and asexual species. Sexuality is based on two compatible mating types and sexual identity is determined by *MAT *loci that encode homeodomain transcription factors, peptide pheromones and their receptors. The objective of the present study was to investigate the presence and integrity of *MAT *genes throughout the phylogenetic diversity of red yeasts belonging to the order Sporidiobolales.

**Results:**

We surveyed 18 sexual heterothallic and self-fertile species and 16 asexual species. Functional pheromone receptor homologues (*STE3.A1 *and *STE3.A2*) were found in multiple isolates of most of the sexual and asexual species. For each of the two mating types, sequence comparisons with whole-genome data indicated that synteny tended to be conserved along the pheromone receptor region. For the homeodomain transcription factor, likelihood methods suggested that diversifying selection acting on the self/non-self recognition region promotes diversity in sexual species, while rapid evolution seems to be due to relaxed selection in asexual strains.

**Conclusions:**

The majority of both sexual and asexual species of red yeasts have functional pheromone receptors and homeodomain homologues. This and the frequent existence of asexual strains within sexual species, makes the separation between sexual and asexual species imprecise. Events of loss of sexuality seem to be recent and frequent, but not uniformly distributed within the Sporidiobolales. Loss of sex could promote speciation by fostering the emergence of asexual lineages from an ancestral sexual stock, but does not seem to contribute to the generation of exclusively asexual lineages that persist for a long time.

## Background

In eukaryotes sexual reproduction is an almost universal trend [1,2], a fact that underlines its importance both for individual fitness and long-term survival of species [3]. Fungi in particular have evolved a rich repertoire of mating strategies and sex-determining systems that has no parallel in other eukaryotic lineages [4-6]. Sexual identity in fungi is always established in the haploid stage [7] and determined by specialized genomic regions called mating type (*MAT*) loci [8]. In general, fungal *MAT *loci encode transcription factors that regulate the expression of determinants of sexual identity/compatibility, such as peptide pheromones and pheromone receptors belonging to the G protein-coupled receptor family [9].

In Ascomycota, and possibly also in Mucoromycotina and Chytridiomycota, the *MAT *loci occur in two alternate forms that determine two mating types or sexual compatibility groups [6,10-12]. This constitutes the so-called bipolar mating system. However, in Basidiomycota, two functional classes of genes can be found within *MAT *loci: in addition to transcriptional regulators (homeodomain transcription factors - HD1/HD2), the genes encoding pheromones and pheromone receptors (P/PR) are also located in the genomic regions that determine sexual identity. This formed the basis for the existence of a more complex mating system in some basidiomycetes. In this system two classes of *MAT *genes (HD1/HD2 and P/PR) are located in two genetically unlinked *MAT *loci (henceforth designated as HD and PR loci, respectively) [9,13]. This system is called tetrapolar, because four different mating types are generated after meiosis. Irrespective of whether the mating system is bipolar or tetrapolar, mating identity and compatibility are generally determined in basidiomycetes at two levels. First, mating and cell fusion occurs when individuals carry different alleles of genes encoding small lipopeptide pheromones and PR [14,15]. Following fusion of compatible partners, progression through the sexual cycle depends on a second compatibility checkpoint that relies on the formation of a heterodimeric homeodomain transcription factor (HD1/HD2) encoded also at the *MAT *locus [16,17]. The homeodomain transcription factor is only active after cell fusion because dimerization is restricted to HD1 and HD2 subunits that originate from genetically different individuals [18,19]. One additional relevant aspect of the tetrapolar system is that *MAT *loci are often highly polymorphic bringing forth species with many allelic forms that can produce up to thousands of mating types [16,20-23]. Remarkably, in basidiomycetes with many mating types (viz. mushrooms), the control of cell-cell attraction and fusion by P/PR signalling has been abandoned [21] but it is still essential for the development and maintenance of the dikaryotic state. In addition, a third *MAT *system, designated pseudo-bipolar, was recently found in members of the red yeast genera *Rhodosporidium *and *Sporidiobolus*, that belong to an early diverged lineage of basidiomycetes [24,25]. In both bipolar and pseudo-bipolar systems only two mating types are found but in the latter some characteristics of tetrapolar systems are also present, like the occasional occurrence of recombination within the bipolar *MAT *locus [24]. Among the diversity of mating systems found in fungi, various types of self-fertility (or homothallism) have also evolved [26]. In those cases, heterothallism (mating occurs only between compatible individuals carrying different *MAT *alleles [16]), is replaced by the ability of a single clone to form sexual structures and reproduce sexually.

In addition to the various modes of sexual reproduction, asexual reproduction is also a conspicuous process of fungal propagation. This suggests that for fungi the advantages of sex, namely the promotion of adaptive evolution and the more efficient elimination of deleterious mutations [27], may in many instances be insufficient to counterbalance its disadvantages, normally associated with energy and time costs of sexuality [28]. The reasons why sexuality can be facultative in fungi seem to be related to the availability of mitotically formed spores and other modes of asexual propagation [29]. However, although the ability to reproduce mitotically in fast and efficient ways acts potentially as a strong evolutionary promoter of asexuality [30], available evidence indicates that asexuality is a derived character. Firstly, asexual species are clearly a minority (approximately 17% of all known fungal species are asexual). Secondly, asexual species very often have phylogenetically close relatives capable of sexual reproduction [31,32].

In this study we focused our attention on a basal lineage of basidiomycetes that is notorious for the co-existence of sexual and asexual species, the red yeasts of the order Sporidiobolales. This order is classified in the subphylum Pucciniomycotina, the earliest diverged lineage of basidiomycetes [33], whose most notable members are the rust fungi. A classical and presently much debated rule in fungal nomenclature imposes that asexual and sexual fungi are classified separately, in different species and genera [34]. For example, in the order Sporidiobolales sexual species are classified in the genera *Rhodosporidium *and *Sporidiobolus *whereas asexual forms are classified in *Rhodotorula *and *Sporobolomyces *[35]. Therefore, the co-existence in the same phylogenetic group of very closely related species of sexual and asexual taxa with similar ecological and physiological properties raises several questions: (i) are asexual species truly asexual having thus no traces of *MAT *genes in their genomes? (ii) alternatively, have asexual species formed recently and therefore still exhibit (traces of) sex related genes? (iii) is mating type imbalance possibly giving rise to asexual lineages? (iv) is asexuality contributing to speciation by giving rise to new species that derive from extant sexual stocks? These questions were addressed in this study by surveying the presence and integrity of *MAT *genes in an expanded set of red yeast species. In order to get some additional insight in the evolution of *MAT *loci in the Sporidiobolales, we also compared gene content and organization around *MAT *genes between opposite mating types and across different species.

## Results and Discussion

### Updated phylogeny of the Sporidiobolales

**Figure 1:**
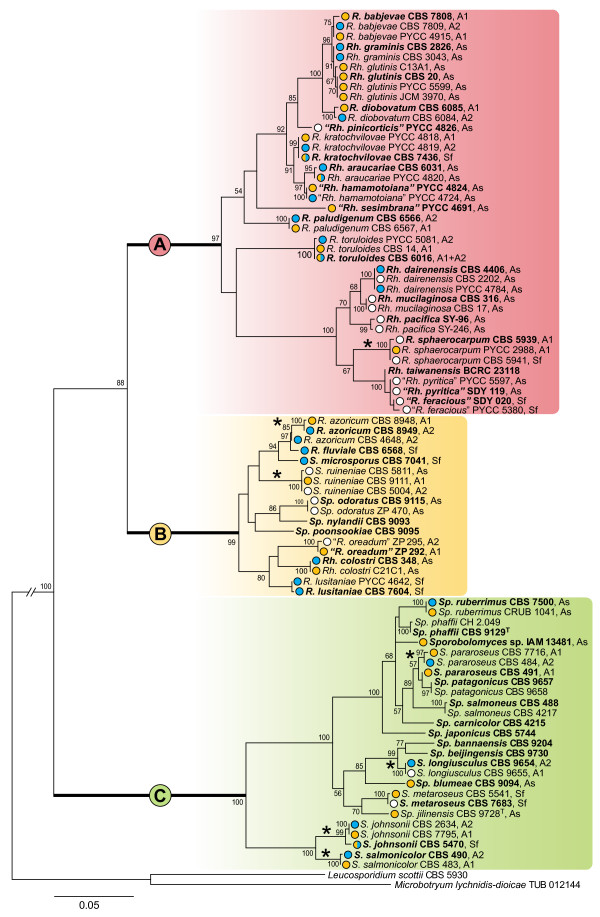
**Phylogeny of the Sporidiobolales**. Molecular phylogeny based on a concatenated alignment of the ITS region (ITS1, 5.8S and ITS2) and the D1/D2 domain of the LSU rRNA. Mating types (*MAT A1 *and *MAT A2*), asexual and self-fertile strains are designated as A1, A2, As and Sf, respectively. Circles before each strain depict the type of PR gene: yellow, *STE3.A1*; blue, *STE3.A2*; white, not detected; half-coloured circles, simultaneous detection of *STE3.A1 *and *STE3.A2*. For each species, the type strain is highlighted in boldface. A, B and C are three statistically well-supported clades (see text for details). Abbreviations of generic names: *Rhodosporidium *(*R*.), *Rhodotorula *(*Rh*.), *Sporidiobolus *(*S*.) and *Sporobolomyces *(*Sp*.). Asterisks indicate instances where the original mating type designation was altered to match the molecular identity of the strains (see Additional File 1). Species not yet formally described are indicated by inverted commas. The tree was rooted with sequences of *Microbotryum lychnidis-dioicae *and *Leucosporidium scottii *(see methods). Branch lengths are given in number of substitutions per site. Bootstrap values (>50%) from 1000 replicates are shown.

We investigated a group of 43 red yeast species representing the phylogenetic diversity in the order Sporidiobolales. These species are classified in four genera, two sexual - *Rhodosporidium *and *Sporidiobolus *- and two asexual - *Rhodotorula *and *Sporobolomyces*. An updated molecular phylogeny of the Sporidiobolales based on the concatenated alignment of the complete ITS region (ITS1, 5.8S and ITS2) and the D1/D2 domain of the LSU rRNA is shown in Figure 1. Three statistically well-supported clades were observed. Although such organization was somewhat anticipated in previous studies (e.g. [35,36]), the incorporation of more taxa, including species not yet formally described, and the combination of ITS and D1/D2 data, improved the resolution of the present analysis. Whereas clade A included only species of *Rhodosporidium *and *Rhodotorula*, which are characterized by the absence of forcibly discharged spores (ballistoconidia), clade C contained only species of *Sporidiobolus *and *Sporobolomyces *that are able to form ballistoconidia. At variance with clades A and C, clade B included representatives of the four genera, which indicates that the traditional classification separating genera based on the ability to form ballistoconidia is not supported at the molecular level. It is also evident from Figure 1 that in all three clades, sexual and asexual species are completely intermingled.

### Pheromone receptor genes as molecular markers of mating type identity

Multiple *HD1/HD2 *alleles were found in red yeast species, and phylogenetic analyses suggested that allele diversification occurred only after each species was formed [24]. In addition, *HD1/HD2 *genes of different red yeast species were found to be highly divergent precluding their utilization for mating type screening purposes over a wide phylogenetic range. On the contrary, phylogenetic analyses of the PR alleles (*STE3.A1 *and *STE3.A2*) of both *M. violaceum *and *S. salmonicolor*, revealed ancient trans-specific polymorphisms [24,37] as expected for genes maintained under long-term balancing selection [38]. Since only two PR alleles were present in each species examined so far, and the presence of each allele correlated in all cases with mating behaviour [24,39], PR genes can be advantageously used as markers for mating type at the molecular level across a broad range of red yeast species.

**Figure 2:**
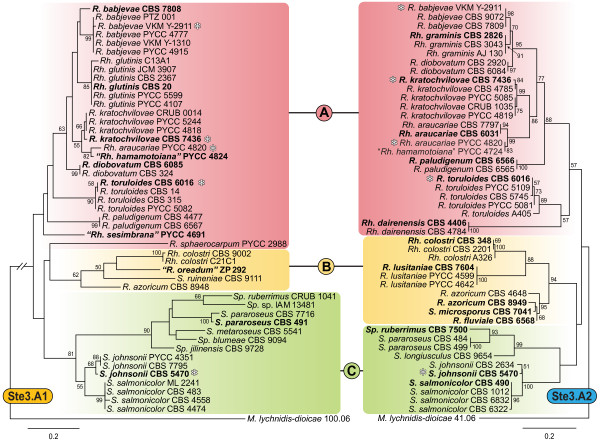
**Phylogeny of the pheromone receptors**. Phylogeny based on the amino acid sequence of the *MAT A1 *and *MAT A2 *PR (Ste3.A1 and Ste3.A2, respectively) of several red yeast species. Groups A, B and C are the same as in Figure 1. Strains marked with asterisks have both PR. *MAT A1 *and *MAT A2 *PR sequences from *Microbotryum lychnidis-dioicae *were used as outgroups. Bootstrap values (>50%) from 1000 replicates are shown. See Figure 1 for additional details.

All heterothallic red yeast species within the order Sporidiobolales were originally described as having a bipolar mating behaviour. Such studies involved only standard crossing tests and strains were therefore arbitrarily assigned to either *MAT A1 *or *MAT A2 *within each species. PR genes were first identified in *R. toruloides *and were named *STE3.A1 *and *STE3.A2 *in accordance with the mating type of the strains in which they were initially characterized [39]. After the identification of the sequences of the PR genes in *S. salmonicolor *and *S. johnsonii*, it became clear that as a result of the original arbitrary assignment of mating types, each *STE3 *allele was associated with strains of the opposite mating type, (e.g. *STE3.A1 *was found in *S. salmonicolor *and *S. johnsonii MAT A2 *strains, [24]). To avoid future confusion, we previously renamed the mating type designations of these two species to match the molecular data [24]. Here we investigated at the molecular level the current mating type designations of the heterothallic species included in this work. To that end we employed a PCR-based approach with degenerate primers that specifically amplified *STE3.A1 *or *STE3.A2 *alleles. In approximately half of the heterothallic species analysed (*R. sphaerocarpum*, *R. azoricum*, *S. ruineniae*, *S. pararoseus *and *S. longiusculus*) the incongruences previously observed for *S. salmonicolor *were also detected, and in these cases the mating type designations were altered so as to match the molecular identity of the strains (Additional File 1). It should be noted that in *R. sphaerocarpum *and *S. ruineniae *only the *STE3.A1 *allele could be identified (Figure 2 and Additional File 1). Nevertheless, the fact that the *STE3.A1 *allele was only found in strains that mated as *MAT A2 *suggested that the *STE3.A2 *allele is most probably present in *MAT A1 *strains, but the set of primers that we used failed to amplify it. For *R. babjevae *we investigated 25 strains (Additional File 1). Previously, mating studies had identified five strains as *MAT A1*, four strains as *MAT A2 *and two strains as self-fertile. The remaining 14 strains were found to be sexually impaired and thus considered asexual. The molecular characterization of PR alleles confirmed for the sexual strains the results obtained with standard crossing tests. It also showed that one of the self-fertile strains (VKM Y-2911) contained both receptor alleles and that 11 asexual strains belonged to *MAT A1 *and three to *MAT A2*. Both mating types were present in strains isolated in Russia (six strains), Argentina (five strains) and Japan (two strains). However, the six strains isolated in Portugal belonged solely to *MAT A1*. Using tester strains with good mating abilities and five asexual strains for which the molecular determination of mating type had been obtained, we repeated crosses and confirmed that these strains do not mate in culture.

### Pheromone receptor genes in asexual species

We subsequently investigated the presence of PR homologues (*STE3.A1 *and *STE3.A2*) in 16 asexual species classified in the genera *Rhodotorula *and *Sporobolomyces *(Figure 1 and Additional File 1). Amplification of *STE3.A1 *and/or *STE3.A2 *alleles was successful in 11 species among the 16 examined (Figure 1). When multiple strains were available for a species, both PR alleles were normally detected, signalling the presence of the two mating types in natural populations of these presumably asexual species. However, an interesting exception was found for the sibling species *Rh. glutinis *and *Rh. graminis*. Whereas all six strains of *Rh. glutinis *possessed the *STE3.A1 *allele, all four strains of *Rh. graminis *exhibited the *STE3.A2 *allele (Figure 2 and Additional File 1). These two species are also closely related to the heterothallic species *R. babjevae*. In contrast to *R. babjevae*, only a limited number of strains of *Rh. graminis *and *Rh. glutinis *have been isolated worldwide [40], all of which were included in the present study. For these three species, inter-species mating has been tested, but was not observed [41]. It is tempting to speculate that asexuality in *Rh. graminis *and *Rh. glutinis *might be related to a strong but relatively recent mating type imbalance. Nevertheless, a larger sampling of both species would be required to accurately determine the mating type distribution and frequency and to elucidate if sex occurs in nature by looking for footprints of recombination, as done previously for other asexual fungi [42]. Moreover, it is important to note that mating type imbalance does not lead necessarily to asexuality as demonstrated in *C. neoformans *[43,44].

Next, we investigated the integrity of PR genes in asexual species. To this end, the complete genes were sequenced. We found that none of them showed hallmarks of degradation and that a protein sequence could be readily obtained by conceptual translation in all cases, suggesting that these genes encode functional proteins. We also inspected the PR genes in the recently released genomes of the presumptively asexual species *Sporobolomyces *sp. IAM 13481 (*STE3.A1*) and *Rh. graminis *WP1 (*STE3.A2*) (formerly designated as *R. babjevae*), available at the Joint Genome Institute (JGI). Again, intact and complete sequences of the PR genes were identified. Therefore, our results suggest that asexuality in red yeasts is not ancient, which is consistent with other studies that have shown that in presumed asexual ascomycetes, mating type genes are present and apparently functional [45,46]. It is also possible that some supposedly asexual species have retained a cryptic sexual stage that is difficult to induce in the laboratory.

In contrast to these observations, for five asexual species (*Rh. mucilaginosa*, *Rh. pacifica*, *"Rh. pinicorticis"*, *"Rh. pyritica" *and *Sp. odoratus*) no PR alleles were found (Figure 1 and Additional File 1). With the exception of *"Rh. pinicorticis" *and *Sp. odoratus*, the remaining species are closely related to each other and belong to a lineage of clade A that includes also *R. sphaerocarpum *and *Rh. dairenensis*. The phylogenetic analysis of *STE3.A1 *from *R. sphaerocarpum *and *STE3.A2 *from *Rh. dairenensis*, suggested that the amino acid sequences of these PR genes are unusually divergent (Figures 1 and 2). Therefore, it is conceivable that the lack of amplification in the remaining species is due to more divergent but still functional sequences. A similar explanation might apply for the amplification of *R. sphaerocarpum STE3.A1 *in only one out of six *MAT A1 *strains investigated.

For *Rh. araucariae *PYCC 4820 two PR alleles (*STE3.A1 *and *STE3.A2*) were detected (Figures 1 and 2, Additional File 1). However, the nucleotide sequence of the *STE3.A2 *allele in this strain differed from homologous sequences of conspecific strains of the same mating type (CBS 6031 and CBS 7797; 87% identity). Surprisingly, the *STE3.A2 *allele of *Rh. araucariae *PYCC 4820 was found to be 100% identical to the *STE3.A2 *gene of *"Rh. hamamotoiana" *PYCC 4724. Contrary to the other strains of *Rh. araucariae*, cells of strain PYCC 4820 formed a slender projection that resembled a conjugation tube, although actual conjugation between cells of the same strain or with cells of the other strains of *Rh. araucariae *was not observed. It seems plausible that the phenotype of PYCC 4820 is due to the presence of *MAT *alleles of both mating types (including the P/PR), as a result of a past hybridization event between the two species. However, incompatibilities at other loci, like for example the HD locus, are perhaps impairing progression through the sexual cycle, as no dikaryotic mycelium or teliospores were observed.

### Pheromone receptor genes in self-fertile species

In homothallic species, a single strain can complete the life cycle without the need for a complementary mating partner. Although several mechanisms of homothallism have been put forward in fungi [26], the most common explanation for this sexual behaviour is that these species have both *MAT *alleles in a single genome and can therefore form sexual structures alone. In this study we searched for the presence of both PR alleles in three species from clade B that contain only self-fertile strains - *R. fluviale*, *R. lusitaniae *and *S. microsporus *- but only *STE3.A2 *alleles were found. Although we could not rule out that the absence of amplification of *STE3.A1 *alleles was due to mutations in primer target sequences, we deem this explanation less likely in this case, since *STE3.A1 *alleles were identified and sequenced in closely related species of clade B, like *R. azoricum *and *Rh. colostri*. We hypothesize that for these three species self-fertility could be due to compatibility at the HD locus despite homozygosity at the PR locus. If confirmed, such a situation would parallel that found in the corn smut pathogen *Ustilago maydis*, in which diploid strains that are homozygous at the PR locus but heterozygous at the HD locus are able to produce teliospores that germinate and undergo meiosis in a way that is indistinguishable from that of the heterozygous diploids at both *MAT *loci [47]. A similar self-fertile phenotype is originated in *C. neoformans *when either the *α *sex inducer gene *SXI1α *(HD1) is introduced into *a *cells or the *a *sex inducer gene *SXI2a *(HD2) is introduced into *α *cells [48,49]. Nevertheless, whereas the diploid strains of *U. maydis *only produce incipient hyphae because maintenance of filamentous growth also requires heterozygosity at the PR locus [47], for the self-fertile yeast species studied we found extensive hyphal growth. Therefore, this also raises the possibility that in self-fertile red yeast species, the PR locus may be self-compatible either by constitutive activation of the pheromone receptor or by an autocrine signalling loop to produce a permanent signalling pathway. Interestingly, examples of mutations supporting both hypotheses have been reported in laboratory [50-52]. Some heterothallic species of red yeasts also include a few self-fertile strains (viz. *R. kratochvilovae *CBS 7436, *R. babjevae *VKM Y-2911 and *S. johnsonii *CBS 5470). In most of those cases we found that both PR alleles were present (Additional File 1) suggesting that self-fertility in red yeasts can be attained by different molecular mechanisms.

### Phylogenies of pheromone receptors

Using partial sequences of *STE3.A1 *and *STE3.A2 *alleles we compared the evolutionary history of the two PR whose trans-specific phylogenies indicate that their divergence dates back to the origin of sexuality in basidiomycetes [24,37,53]. Phylogenies based on the protein sequences of the PR genes were constructed separately for each receptor (Ste3.A1 and Ste3.A2) in order to maximize the quality of the alignments (Figure 2). In spite of general concordance between rDNA and PR trees, three conflicts were detected in the Ste3.A1 tree. These cases correspond to the placement of *R. diobovatum*, *R. paludigenum *and *R. sphaerocarpum *outside their respective groups, by comparison with rDNA and Ste3.A2 trees (Figures 1 and 2). However, it should be noted that bootstrap values in the Ste3.A1 tree tended to be lower than those obtained for the Ste3.A2 tree and that therefore these conflicts might be due to poor resolution in the Ste3.A1 tree.

### Structure of the *MAT A1 *and *MAT A2 *genomic regions encompassing the alternate pheromone receptors

**Figure 3:**
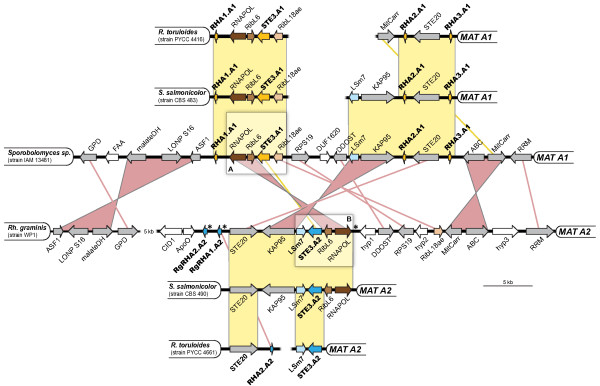
**Synteny between *MAT A1 *and *MAT A2 *PR loci in different species**. The structure of the *MAT A1 *and *MAT A2 *PR loci (~50 kb) is shown for *Sporobolomyces *sp. IAM 13481 and *Rhodotorula graminis *WP1 and compared with shorter homologous regions of *S. salmonicolor *and *R. toruloides. MAT A1 *and *MAT A2*-specific genes are depicted in yellow and dark blue arrows, respectively, showing the direction of transcription. Genes enclosed in box A are flanking the *STE3.A1 *gene in most members of Sporidiobolales and those in box B are usually flanking the *STE3.A2 *gene. Orthologous genes in boxes A and B have the same colour. Genes shared by *Sporobolomyces *sp. and *Rh. graminis *are shown in gray, while others are shown in white. Yellow lines or bars connect, respectively, genes or syntenic blocks that are in the same orientation, while pink lines or bars indicate inversions. Asterisks in the *MAT *region of *Rh. graminis *indicate three identical repeats of ~280 bp (the repeat located downstream of the *RNAPOL *gene is inverted in relation to the other two).

We have previously observed in a restricted number of red yeast species that genomic regions around the PR locus exhibited synteny breaks, gene inversions and sequence divergence between the two mating types as a result of apparent partial suppression of recombination, while synteny was well conserved across species when *MAT *regions of the same mating type where compared [24,39]. Here we set out to assess whether these observations also held true for the expanded set of red yeast species presently under scrutiny. Taking advantage of whole-genome data of *Sporobolomyces *sp. IAM 13481 (*MAT A1*) and *Rh. graminis *WP1 (*MAT A2*), we assessed the synteny around the PR genes in red yeast species belonging to clades A, B and C (Figure 1 and Additional File 2). PCR primers anchored on the 3' end region of the genes flanking the *STE3.A1 *(*RibL18ae *and *RibL6*/*RNAPOL*) and *STE3.A2 *(*LSm7 *and *RibL6*/*RNAPOL*) yielded almost always an amplification product between 2.5 to 3.0 kb, consistent with a gene organization identical to that found in the genomes of *Sporobolomyces *sp. IAM 13481 and *Rh. graminis *WP1 (Figure 3). Sequencing of the complete intervening regions confirmed the suspected synteny within the same mating type across the majority of the species (Additional File 2). However, for some species, like *MAT A1 *strains of *R. paludigenum*, gene order seemed to have been disrupted since no amplification products were obtained even when using different combinations of primers for the more conserved flanking genes. Another interesting exception was *S. ruineniae *CBS 5811, where a *MAT A2 *pheromone precursor homologue similar to the *RHA2.A2 *of *R. toruloides *[39] was found between *RibL18ae *and *RibL6 *instead of the *STE3.A1 *gene usually present at this location (Additional File 2). This indicates that the *STE3.A1 *gene has been relocated to a different and presently unknown genomic location in this species.

We next examined a larger genomic region of the *MAT A1 *and *MAT A2 *loci. To do so, 50 Kb regions encompassing the *STE3.A1 *gene in *Sporobolomyces *sp. IAM 13481 and the *STE3.A2 *gene in *Rh. graminis *WP1 were compared (Figure 3). In *Rh. graminis *WP1 we identified two identical putative pheromone precursor homologues (Rg*RHA1.A2 *and Rg*RHA2.A2*), similar but not syntenic to the *RHA2.A2 *gene of *R. toruloides MAT A2 *strains (Figure 3) [39]. The regions upstream of Rg*RHA2.A1 *and Rg*RHA2.A2 *were identical. A shorter sequence of approximately 280 bp was also identical and was detected downstream of *RNAPOL *in an inverted orientation (Figure 3). This suggests that an additional copy of the pheromone precursor gene may have existed at this genomic location similarly to that found in *MAT A1*. Notably, *MAT A1 *pheromone genes in *S. salmonicolor *and *R. toruloides *were found to be embedded in conserved inverted repeats [39], a situation earlier documented in the basidiomycete human pathogen *C. neoformans *[54]. These repeats are likely maintained by intra-allelic gene conversion to ensure maintenance of gene function in face of the absence of meiotic recombination characteristic of these regions [55,56].

Our comparison of the gene content of the *MAT *genomic regions shows that gene content is similar despite the phylogenetic distance between *Sporobolomyces *sp. and *Rh. graminis *(Figures 1 and 3) and that apparently functional copies of each allele have been retained in both *MAT *loci. This is in contrast to genes encoded in sex chromosomes of diploid organisms, for which degeneration and loss of one functional copy frequently occurs [57]. Maintenance of both alleles in fungal sex-determining regions, including the *MAT *loci of red yeasts, is most probably related to the fact that fungi commonly occur as haploids in the environment, and thus gene degeneration or loss is very likely to be detrimental. However, gene order seems to have been deeply altered during mating type divergence and many gene blocks were found in inverted positions, probably restraining recombination between opposite *MAT *regions (Figure 3). On the contrary, within the same mating type, the analysed regions were found to be highly syntenic even between more distantly related species (Figure 3).

### Diversity and evolution of homeodomain transcription factors

**Figure 4:**
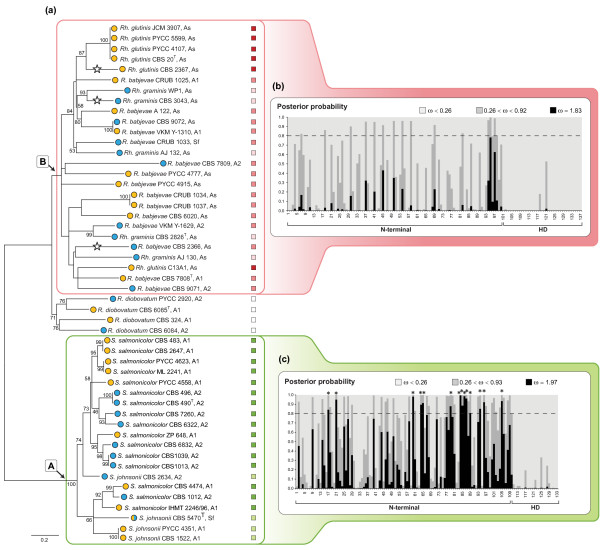
**Diversity of *HD1/HD2 *alleles and evolution of *HD1 *in two different clades of red yeast species**. **(a) **Phylogeny of the *HD1/HD2 *alleles in two groups of red yeasts. Abbreviations of generic names are as in Figure 1. Strains belonging to the same species are indicated by boxes of the same colour after strain numbers: Clade A - *S. salmonicolor *(green) and *S. johnsonii *(light green); clade B - *Rh. glutinis *(red), *R. babjevae *(pink) and *Rh. graminis *(light pink). *R. diobovatum *formed a more divergent group external to clade B. Sexual (*MAT *A1/A2), asexual and self-fertile strains are indicated as "A1/A2", "As" and "Sf", respectively, after strain numbers. Circles before each strain depict the type of pheromone receptor genes (yellow, *STE3.A1*; blue, *STE3.A2*). White stars in tree branches symbolize premature stop codons detected in the corresponding *HD2 *alleles. Branch lengths are given in number of substitutions per site. Bootstrap values (>50%) from 1000 replicates are shown. **(b) **Posterior probabilities of site classes for codons along the N-terminal and HD domains of the *HD1 *gene, obtained under model M8 (beta&ω) for the clade B dataset. The first six categories are collapsed into one, represented as ω < 0.26 (purifying selection) as well as the categories seven to ten that are represented as 0.26 < ω < 0.92. **(c) **Posterior probabilities obtained for the dataset of clade A under the model M8. The first 5 categories are collapsed into one (ω <0.26) as well as the categories six to ten (0.26 < ω < 0.93). Sites for which the posterior probability for the site class of positive selection (with ω > 1) exceeds 0.80 are inferred to be under diversifying selection and are indicated by asterisks (see Supplementary Tables S1 and S2 in Additional File 4 for details).

We have previously observed that the *HD1/HD2 *alleles of some red yeasts are much more divergent than the correspondent PR genes [24], and therefore less suitable for comparisons involving more distantly related species. Nevertheless, we aimed to ascertain if the genes encoding these transcriptional regulatory factors appear to be functional within a more expanded group of red yeasts including sexual and asexual strains and species and to evaluate their pattern of evolution. To this end, partial sequences of *HD1/HD2 *alleles were obtained for six species and their phylogeny is shown in Figure 4. In clade A, formed by *S. johnsonii *and *S. salmonicolor*, the topology of the *HD1/HD2 *tree conflicts with the rDNA (Figure 1) and Ste3 (Figure 2) trees because *S. johnsonii *CBS 2634 harbours an *HD1/HD2 S. salmonicolor*-type allele and the converse situation occurs for *S. salmonicolor *strains CBS 1012, CBS 4474 and IHMT 2446/96 (Figure 4a). Such discrepancies suggest that the two species are not fully genetically isolated and that mating with exchange of *HD1/HD2 *alleles is still possible. In clade B, the lack of strict species-specificity of *HD1/HD2 *alleles is also evident for the complex *R. babjevae - Rh. glutinis - Rh. graminis*. Our observation that pheromone precursor genes seem to encode very similar or even identical peptides in closely related species, possibly precluding discrimination between species prior to cell fusion is also in line with this. For example, only synonymous substitutions were detected when analyzing the *RHA2.A1 *genes encoding the tandem pheromone peptides from different strains of *S. salmonicolor *and *S. johnsonii *(Additional File 3). This results in the production of identical peptide pheromones, a circumstance that is likely to facilitate interspecies mating.

Judging from available data, a geographic structure for *HD1/HD2 *allele distribution seems to be lacking since strains of *Rh. glutinis *from different regions have the same *HD1/HD2 *alleles and in *Rh. graminis *different alleles were recovered from the same region (Figure 4a and Additional File 1). For most of the species studied each strain had a unique *HD1/HD2 *allele although in *Rh. glutinis *four strains shared the same *HD1/HD2 *allele. For two strains of *R. babjevae *(VKM Y-1310 and CBS 9072) it seems that a recent recombination event between the two *MAT *regions gave rise to strains with different PR alleles that share the same *HD1/HD2 *allele, consistent with the proposed pseudo-bipolar system [24] (Figure 4a). Although the phylogenies and diversity of *HD1/HD2 *alleles in *S. salmonicolor *and *S. johnsonii *suggested that such events were likely to occur albeit at low frequency, they had been thus far detected only in laboratory crosses [24].

Allele divergence as inferred by branch length seems to be lower for the species in clade A, solely composed of sexually competent strains, than in clade B, which is dominated by asexual strains (Figure 4a). Adaptive changes in protein-coding genes may be detected by comparing the number of synonymous (d_S_) and nonsynonymous (d_N_) substitutions per site, with the ratio ω = d_N_/d_S _providing a measure of selection at the protein level. We used seven codon-site models of variable ratios of ω across site, as implemented in PAML version 4.0 [58], to investigate the evolutionary constraints acting on the N-terminal domain of *HD1 *alleles, which is known to define mating specificity, as well as the initial part of the homeodomain motif. This analysis was done separately for each clade, and for clade B it involved sexual and asexual species and sexual and asexual strains within a species (*R. babjevae*), whereas clade A was composed solely of sexual strains (Figure 4). Detailed descriptions of the models, likelihood ratio statistics and parameter estimates for each dataset are given in Additional File 4. *R. diobovatum *was excluded because it was too divergent from the remaining species in clade B, presenting d_S _> 2.0 in most pairwise comparisons. We found that the *HD1 *gene evolves faster in clade A than in clade B given the averaged values of ω for the two clades (0.504 vs. 0.172, respectively) obtained from model M0 (one ratio), which assumes a single ω for all codons in the sequence. However, synonymous substitution rates were significantly higher in clade B than in clade A (*P *< 0.001, Mann-Whitney U-test), indicating that in clade B, the *HD1 *gene may be under fewer functional constraints, possibly due to a lower strength of codon usage bias associated with changes in the level of gene expression [59]. For the dataset of clade A, models M2a (selection) and M8 (beta&ω), which allow for positive selection at a subset of sites, fitted the data better than the null models M1a (nearly-neutral), M7 (beta) and M8a (beta&ω_s_= 1), which do not allow for positive selection (Supplementary Tables S1 and S2 in Additional File 4). Parameter estimates under model M8 (beta&ω) for the dataset of clade A suggest that ~ 22% of the sites are under diversifying selection (ω > 1) with ω_s _= 1.97. These sites were found within the regions determining *MAT *specificity, in accordance with previous observations [24]. Nevertheless, the average ω along the N-terminal region was < 1, as also observed in other systems such as the *S*-loci of plants [60,61]. Additionally, sites within the homeodomain motif exhibited low ω (ω < 0.26) indicative of strong purifying selection. The posterior probabilities obtained under model M8 for the different site classes (see Supplementary Table S1 in Additional File 4) are shown in Figure 4. For the clade B dataset (*R. babjevae*, *Rh. glutinis *and *Rh. graminis*), positive selection models did not fit the data better than the models implying neutral evolution indicating that positive selection is not driving the evolution of the *HD1 *gene in this clade (see Supplementary Table S2 in Additional File 4). Moreover, model M8a (beta&ω_s_= 1) where an extra site class was constrained to have ω = 1, seems to fit the data better than other models (Supplementary Table S2 in Additional File 4) consistent with the identification of relaxed purifying selection [62]. This may be a consequence of loss of sexual competence in *Rh. glutinis*, *Rh. graminis*, and in several strains of *R. babjevae*. In line with this, stop codons were found in N-terminal regions of the *HD2 *gene of *Rh. graminis *CBS 3043 and *R. babjevae *CBS 2366, which is asexual. In *Rh. glutinis *CBS 2367 a dinucleotide repeat (CT)_13_ insertion followed by a premature stop codon most likely renders this gene non-functional. Although C-terminal truncations of other HD proteins, including Sxi1α in *C. neoformans *[44], did not affect their functionality, the early stop codons found in asexual strains of red yeasts are located in the N-terminal region of the protein, thus presumably originating very short (non-functional) proteins devoid of homeodomain motifs. For the *HD1 *gene, no stop codons were found in the analysed regions and the evolution of the homeodomain motif seems to be dominated by purifying selection in species of both clades. While this may suggest that HD1 proteins have additional roles that do not require heterodimerization (a possibility so far poorly explored in basidiomycetes), another possibility is that this gene is losing its mating specific role in species of clade B as a consequence of relaxed functional constraints. Therefore, it seems possible that the sequence divergence of the *HD1/HD2 *genes examined here is governed both by diversifying and by relaxing selection, and that this may be either the cause or the consequence of loss of sexual competence by individual strains.

Our results suggest that loss of sex might not occur uniformly across the Sporidiobolales. Different molecular evolution patterns may be related with distinct selective constraints acting on different species. *S. salmonicolor *and *S. johnsonii *exemplify a case of strictly sexual red yeasts whose *HD1/HD2 *alleles exhibit low d_S_ values possibly due to some form of constraint on synonymous substitutions like codon bias [63,64] (Figures 4), but where allele specific regions are evolving under positive selection. The study of maintenance or loss of sex in red yeast could benefit from a larger population sampling that would allow to look for genetic footprints of recombination [42]. Nevertheless, even fungi shown to be purely clonal from a population genetic perspective (viz. *Penicillum marneffei*), also present *MAT *genes in their genomes [65], suggesting that sex has been lost only recently.

## Conclusions

We examined 216 strains belonging to 32 species of the Sporidiobolales and found apparently functional PR homologues for most strains, irrespective of their sexual or asexual status. In addition, we observed that all asexual species have phylogenetic close relatives that mate well in laboratory conditions and we think it is likely that asexual red yeasts have lost the ability to cross, although this has not (yet) resulted in the degeneration or loss of *MAT *genes in most cases. However, we detected mutations likely driving loss of function in *HD2 *homologues in some asexual strains here investigated, which seems to be consistent with relaxed selection as a consequence of loss of sex.

Taken together, our results suggest a fuzzy separation between sexual and asexual red yeast species, illustrated by the existence of asexual strains within sexual species and by the conservation of *MAT *regions in asexual species. The co-existence of sexual and asexual reproduction in red yeasts probably facilitates the emergence of asexual lineages. For example, *Rh. glutinis *and *Rh. graminis *seem to derive from populations of a heterothallic *R. babjevae*-like ancestor that have independently lost the ability to mate. Notably, sexual reproduction in the latter species seems to be lost easily, as many of the *R. babjevae *strains we investigated were unable to mate.

Several authors have argued that most asexual fungi are evolutionary dead ends awaiting extinction [29,66,67]. This fits well with our observations in red yeasts since we did not find evidence among extant species for ancient events of loss of sexuality. Instead, we uncovered abundant substantiation for frequent and recent such events, leading in some cases to the isolation of lineages and eventually to speciation but never to the generation of exclusively asexual phylogenetic lineages that persist for a long time.

## Methods

### Strains and mating tests

The list of strains studied and relevant information pertaining to them is given in Additional File 1. To study sexual compatibility, pairs of 2-4 day-old cultures were mixed on corn meal agar (Difco), incubated at 18 °C - 22 °C for 1 week and examined microscopically using phase-contrast optics for production of mycelium with clamp connections and teliospores (globose and thick-walled resting structures that are the site of karyogamy).

### PCR detection and sequencing of *MAT A1 *and *MAT A2 *pheromone receptor genes and the conserved surrounding regions

Diagnostic PCRs with degenerate primers for *STE3.A1 *and *STE3.A2 *alleles were carried out to directly identify the PR genes and (re)assign the molecular mating types in most of the studied red yeast strains (Additional Files 1 and 5). For some selected strains the amplification products were purified and sequenced with the same primers (Additional File 1). However, this approach failed to yield amplification products for some of the species. In these cases, *STE3.A1 *and *STE3.A2 *alleles were detected by long range PCR using primers based on the available sequences of the more conserved genes flanking the *STE3.A1 *in *Sporobolomyces *sp. IAM 13481 (genes *RibL18ae *and *RibL6 *or *RNAPOL*) and *STE3.A2 *in *Rh. graminis *WP1 (genes *LSm7 *and *RibL6 *or *RNAPOL*) (Additional File 5). The amplification products were subsequently sequenced by primer-walking (Additional File 5). This latter approach was also used to confirm if synteny in the immediate vicinity of the PR genes in *MAT A1 *and *MAT A2 *strains was maintained across an expanded set of red yeast species (Additional Files 2 and 5). For each yeast species, PCR reactions, cycling conditions and primer sequences are specified in Additional File 5.

### PCR amplification and sequencing of HD1/HD2 region and the *RHA2 *pheromone precursor gene

Using the *HD1/HD2 *sequences of *S. salmonicolor *and *Sporobolomyces *sp. IAM 13481 [24], a Blastn search was performed in the NCBI Trace Archive database of *Rh. graminis *WP1 and the sequences corresponding to positive hits were assembled. The genome of *Rh. graminis *WP1 http://genome.jgi-psf.org/Rhoba1_1/Rhoba1_ 1.home.html was employed to confirm the assembled region, which is located in scaffold 16 (base positions 15900-16300). The deduced HD1 and HD2 protein sequences of *Rh. graminis*, *S. salmonicolor *and *Sporobolomyces *sp. IAM 13481 were aligned and the conserved regions were used to design degenerate primers (MC118 and MC120) to amplify and sequence the corresponding N-terminal and intergenic regions of the *HD1/HD2 *genes in several strains of *R. babjevae*, *Rh. graminis*, *Rh. glutinis *and *R. diobovatum *(Additional Files 1 and 5). GenBank accession numbers are listed in Additional File 1. The *RHA2 *gene was amplified in selected *S. salmonicolor *strains using primers (MC040 and MC073) that anchored on the *STE20 *and *KAP95 *flanking genes. The amplification products were subsequently sequenced by primer-walking. For PCR reactions, cycling conditions, primer sequences and GenBank accession numbers see Additional File 5.

### Sequence data and phylogenetic analyses

For the species tree, PCR amplification and sequencing was done as previously described [41]. Sequences of both regions were concatenated and subsequently aligned using ClustalW 1.4 [68] included in the BioEdit software [69]. The phylogenetic tree was inferred by Maximum Likelihood (ML) with PhyML [70]. FindModel, a web implementation of ModelTest [70,71], was used with the Akaike information criterion (AIC) to select the model that best fit our data. The General Time Reversible model with a discrete gamma distribution was chosen (GTR+G; shape parameter = 0.1947). *Microbotryum lychnidis-dioicae *(DQ366868/AY877416) and *Leucosporidium scottii *(AF0700419/AF444495) were used to root the tree. For the *HD1/HD2 *tree (Figure 4), nucleotide sequences were aligned with MUSCLE [72] since it produced a better alignment for such highly divergent sequences. The phylogenetic tree was also inferred by ML based on the TN93+G+I model (shape = 2.0446; pinv = 0.0524). Protein sequences of the PR (Ste3A1 and Ste3A2) were deduced from the DNA sequences after removal of putative introns, either manually or using AUGUSTUS software [73]. ProtTest [74] with Akaike information criterion (AIC) was used to select the model that best fit our data and phylogenetic trees were inferred with maximum likelihood (ML). The MtREV+G+F model (shape parameter = 1.198) [75-77] was chosen for the Ste3A1 tree and the WAG+G+F (shape parameter = 1.0496) [76-78] was selected for the Ste3A2 dataset. *Microbotryum lycnhidis-diocae *pr-MatA1 (EF584742) and pr-MatA2 (ABU62846) were used to root Figure 2 trees. Bootstrap values were calculated from 1000 replicates for all trees. GenBank accession numbers of the novel sequences are listed in Additional File 1 and raw phylogenetic data (alignments and tree files) is included in Additional File 6.

### Estimation of the evolution rates of 5' end and homeodomain regions of the *HD1 *gene

The region of the *HD1 *gene corresponding to the 5'end and the initial part of the homeodomain motif was amplified and sequenced for the strains indicated in Additional File 1, which lists also GenBank accession numbers. The deduced protein sequences were first aligned with MUSCLE [72] and then poorly aligned regions and regions containing indels were removed. The resulting alignment was used to obtain the corresponding nucleotide (codon-based) alignment. Likelihood-based tests were used to investigate the type of evolutionary pressure acting on the sequenced region of the *HD1 *gene, using the CODEML program within PAML software (version 4.4) [58]. The analyses were carried out using two separate datasets corresponding to clade A (*S. salmonicolor *and *S. johnsonii*, sexual) and clade B (*Rh. glutinis *and *Rh. graminis*, asexual; *R. babjevae *and *R. diobovatum*, sexual but several strains of the former are asexual). Synonymous substitution rates (d_S_) were estimated for each dataset by comparing rates between taxa in all possible combinations (runmode = -2 in PAML). Since d_S_ values obtained from *R. diobovatum *were > 2.0 in several pairwise comparisons, strains of this species were removed from subsequent analyses. Mann-Whitney U-test was used to examine whether or not the d_S_ pattern differed between both clades. The tree files used as an input file for CODEML were produced by Maximum Likelihood (ML) with PhyML [70] and using FindModel with AIC to select the model that best fit each dataset [T92+G (shape parameter = 1,085) for clade A and TN93+G+I (shape = 1.135; pinv = 0.164) for clade B].

Codon-based likelihood analysis was conducted under seven site models [79,80] implemented in CODEML, and their main characteristics are described in Supplementary Methods (Additional File 4). To verify which models fit the data better, likelihood ratio tests (LRTs) were performed by comparing twice the log-likelihood difference (-2 two nested models usingΔ*l*) between two nested models using a χ^2 ^distribution, with the number of degrees of freedom (df) equal to the difference in the number of parameters between models [81]. To examine whether or not the analysed region evolves under positive selection, models that allow a class of codons with positively selected sites (i.e. d_N_/d_S _or ω > 1 in models M2a and M8) were compared with their nested neutral models (M1 and M7, respectively), using 2 df. [81,82]. In addition, comparison between models M8 and M8a (1 df) also allowed testing for evidence of positive selection and to eliminate the potential identification of relaxed purifying selection [80] (see Supplementary Methods in Additional File 4). According to the LRTs, when the positive selection models fitted the data significantly better that the neutral models, the identification of sites evolving under positive selection was obtained by Bayes empirical Bayes (BEB) calculation of posterior probabilities (pp) for site class implemented in models M2a and M8 [83]. Codon sites with ω > 1 and ω < 0.25 and pp values > 80% were considered to be under positive or purifying selection, respectively. Likelihood ratio statistics and parameter estimates for the two datasets are listed in Supplementary Tables S1 and S2 (Additional File 4).

## Authors' contributions

MAC, PG and JPS conceived the study and designed the experiments. MAC performed the experiments. MAC, PG and JPS analyzed and interpreted the data and wrote the paper. All authors have read and approved the final manuscript.

## Supplementary Material

Additional file 1 **List of species/strains used in this study and relevant information pertaining to them**. GenBank accessions numbers are given for each genomic region analysed in this study. Sequences retrieved from the CBS culture collection database ('CBS seq.') or from genome projects ('JGI genome') are indicated. Mating behaviour as originally determined by classical mating tests ('old') and as reassigned in this study based on the molecular data ('new') is indicated when needed: A1, A2, As and Sf stand for mating type A1, A2, asexual and self-fertile strains, respectively. The "molecular mating type" as identified by PCR detection of the pheromone receptor alleles *STE3.A1 *and *STE3.A2 *is depicted by yellow and blue circles, respectively. White circles indicate strains for which PCR detection gave negative results and 'n.d.' stands for 'not determined'. Strains highlighted in boldface were used in Figure 1 and those indicated by thick-lined blue or yellow circles were used in Figure 3. Sequences accession number of the *HD1/HD2 *alleles used in Figures 4b and 4c are also highlighted in boldface. Abbreviations: *Rhodosporidium *(R.), *Rhodotorula *(Rh.), *Sporidiobolus *(S.), *Sporobolomyces *(Sp.), Type strain (T), Lectotype (LT), Authentic strain (AUT). Species not yet formally described are shadowed in light yellow and those for which no molecular mating type was determined are shadowed in gray. Click here for file

Additional file 2 **Synteny of the genomic regions flanking the alternate PR in *MAT A1 *and *MAT A2 *strains of several red yeasts species**. (a) Simplified illustration of the tree represented in Figure 1, indicating the phylogenetic placement of the species where gene organization in the vicinity of the pheromone receptor genes was determined. Clades A, B and C are the same as in Figure 1 and 2. (b) The mating behaviour (A1, A2, As and Sf stands for mating type A1, A2, asexual and self-fertile, respectively), the molecular mating type (*STE3.A1*, yellow circles; *STE3.A2*, dark blue circles; *STE3.A1 *and *STE3.A2*, half-coloured circles) and the obtained genomic regions flanking the pheromone receptor alleles (*STE3.A1 *and *STE3.A2*) are shown for each strain. Orthologues are shown in the same colour. In *Rhodosporidium lusitaniae*, the intervening region between the *STE3.A2 *and the *RibL6 *genes (faint line) was not sequenced. Abbreviations of generic names are as in Figure 1 and the remaining features are represented as in Figure 3. Click here for file

Additional file 3 **Pheromone precursors of different red yeast species**. (a) The organization of the genomic region encompassing the *MAT A1 *pheromone precursor genes in *Sporobolomyces *sp. IAM 13481 is shown on top. Coding regions of the pheromone precursor genes (*RHA1*, *RHA2 *and *RHA3*) are depicted by gray arrows, indicating the direction of transcription. (b) Alignment of the Rha2 pheromone precursor of different *MAT A1 *red yeast species/strains (Ss, *Sporidiobolus salmonicolor*; Sj, *Sporidiobolus johnsonii*; Sp, *Sporobolomyces *sp. IAM 13481; Rt, *Rhodosporidium toruloides*). Amino acids differing from the *S. salmonicolor *strain CBS 483 are shown in red. Sequence repeats proposed to represent the peptide moiety of the mature pheromone are shadowed and those resembling the CAAX motif are underlined. (c) Phylogenetic tree showing the relationships between *RHA2 *genes from the indicated red yeast species, based on the alignment of their coding sequences. Groups are the same as in (b). Sequences of strains depicted in boldface are shown in (b). The tree was inferred using Maximum Parsimony. Bootstrap values from 1000 replicates are shown in the tree nodes. Click here for file

Additional file 4 ***Supplementary Table S1***. Likelihood ratio statistics and parameter estimates for the dataset of clade A (*S. salmonicolor *and *S. johnsonii*) as inferred under seven models of ω over codons. ***Supplementary Table S2***. Likelihood ratio statistics and parameter estimates for the dataset of clade B (*R. babjevae*, *Rh. glutinis *and *Rh. graminis*) as inferred under seven models of ω over codons. ***Supplementary Methods***. Model characteristics and parameters for CODEML ctl file. Click here for file

Additional file 5 **List of primers and specific PCR conditions used to amplify the indicated regions**. Click here for file

Additional file 6 ***Supplementary Files 1***. Files used to construct the phylogenetic tree represented in Figure 1 (alignment file: "Fig1Align.fas"; Tree files: "Fig1TreeNewick.nwk ", "Fig1TreeNexus.nex", and "Fig1TreeRootNexus.nex"). ***Supplementary Files 2***. Files used to construct the phylogenetic trees represented in Figure 2 (alignment file for Ste3a1: "Fig2A1Align.fas"; Tree files: "Fig2A1TreeNewick.nwk", "Fig2A1TreeNexus.nex", "Fig2A1TreeRootNexus.nex"; alignment file for Ste3a2: "Fig2A2Align.fas"; Tree files: "Fig2A2TreeNewick.nwk", "Fig2A2TreeNexus.nex", "Fig2A2TreeRootNexus.nex"). ***Supplementary Files 3***. Files used to construct the phylogenetic tree represented in Figure 4 (alignment file: "Fig4Align.fas"; Tree files: "Fig4TreeNewick.nwk", "Fig4TreeRootNexus.nex"). Nexus files can be viewed by Mesquite software http://mesquiteproject.org/mesquite/download/download.html). Click here for file
