# Supplementary material for: Evidence for maintenance of sex determinants but not of sexual stages in red yeasts, a group of early diverged basidiomycetes
Source: BMC Evol Biol. 2011 Aug 31;11:249. doi: 10.1186/1471-2148-11-249 (PMC3236058; doi:10.1186/1471-2148-11-249)
Supplement: Additional file 1 — List of species/strains used in this study and relevant information pertaining to them. GenBank accessions numbers are given for each genomic region analysed in this study. Sequences retrieved from the CBS culture collection database ('CBS seq.') or from genome projects ('JGI genome') are indicated. Mating behaviour as originally determined by classical mating tests ('old') and as reassigned in this study based on the molecular data ('new') is indicated when needed: A1, A2, As and Sf stand for mating type A1, A2, asexual and self-fertile strains, respectively. The "molecular mating type" as identified by PCR detection of the pheromone receptor alleles STE3.A1 and STE3.A2 is depicted by yellow and blue circles, respectively. White circles indicate strains for which PCR detection gave negative results and 'n.d.' stands for 'not determined'. Strains highlighted in boldface were used in Figure 1 and those indicated by thick-lined blue or yellow circles were used in Figure 3. Sequences accession number of the HD1/HD2 alleles used in Figures 4b and 4c are also highlighted in boldface. Abbreviations: Rhodosporidium (R.), Rhodotorula (Rh.), Sporidiobolus (S.), Sporobolomyces (Sp.), Type strain (T), Lectotype (LT), Authentic strain (AUT). Species not yet formally described are shadowed in light yellow and those for which no molecular mating type was determined are shadowed in gray. [file 1471-2148-11-249-S1.PDF]

Additional file 1. List of species/strains used in this study and relevant information pertaining to them. GenBank accessions numbers are given for each genomic region analysed in this study. Sequences retrieved from the CBS culture collection database ('CBS seq.') or from genome projects ('JGI genome') are indicated. Mating behaviour as originally determined by classical mating tests ('old') and as now reassigned based on the molecular data ('new') is indicated when needed: A1, A2, As and Sf stands for mating type A1, A2, asexual and self-fertile strains, respectively. The "molecular mating type" as identified by PCR detection of the pheromone receptor alleles *STE3.A1* and *STE3.A2* is depicted by yellow and blue circles, respectively. White circles indicate strains for which PCR detection produced negative results and 'n.d.' stands for 'not determined'. Strains highlighted in **boldface** were used in the figure 1 and those indicated by thick-lined blue or yellow circles were used in figure 2. Sequences accession number of the *HD1/HD2* alleles used in Figures 4b and 4c are also highlighted in **boldface**. Abbreviations: *Rhodosporidium* (*R.*), *Rhodotorula* (*Rh.*), *Sporidiobolus* (*S.*), *Sporobolomyces* (*Sp.*), Type strain (T), Lectotype (LT), Authentic strain (AUT). Undescribed species included in this study are shadowed in light yellow and those for which no molecular mating type was accessed are shadowed in gray.

Species	Strains	D1/D2 LSU (GenBank)	ITS region (GenBank)	Mating behaviour Old	Mating behaviour New	*STE3 A1*	*STE3 A2*	*STE3.A1* (GenBank)	*STE3.A2* (GenBank)	*HD1/HD2* (GenBank)	Isolation source and location
R. babjevae	A122			As		●				**HM143851**	Seawater, South of Portugal
R. babjevae	A125			As		●					Seawater, South of Portugal
R. babjevae	A267			As		●					Seawater, South of Portugal
R. babjevae	A270			As		●					Seawater, South of Portugal
R. babjevae	CBS 322			As		●					Water supply of brewery, Germany
R. babjevae	CBS 2366			As			●			**JN246619**	Air, Japan
R. babjevae	CBS 6020			As		●				**JN246620**	Soil from drill core, Japan
R. babjevae	**CBS 7808**[T]	AF070420	AF444542	A1		●		JN246636		**HM143857**	Herbaceous plant, Moscow, Russia
R. babjevae	**CBS 7809**	AF389828	AF387773	A2			●		JN246573	**HM143856**	Herbaceous plant, Moscow, Russia
R. babjevae	CBS 9071			A2			●			**JN246621**	Barley kernels, North Dakota, USA
R. babjevae	CBS 9072			As			●		JN246575	**HM143852**	Barley kernels, North Dakota, USA
R. babjevae	CRUB 1025			A1		●				**JN246614**	Nahuel Huapi, Patagonia, Argentina
R. babjevae	CRUB 1033			Sf			●			JN246615	Verde Lagoon, Patagonia, Argentina
R. babjevae	CRUB 1034			As		●				**JN246616**	Verde Lagoon, Patagonia, Argentina
R. babjevae	CRUB 1037			As		●				HM143854	Fonk Lake, Patagonia, Argentina
R. babjevae	CRUB 1113			As			●				Verde Lagoon, Patagonia, Argentina
R. babjevae	PTZ 001			A1		●		JN246641			Herbaceous plant, Prioksko-terrasny reserve, Moscow, Russia
R. babjevae	PTZ 111			A1		●					Herbaceous plant, Prioksko-terrasny reserve, Moscow, Russia
R. babjevae	PYCC 4651			As		●					Leaf, Estoril, Portugal
R. babjevae	PYCC 4777			As		●		JN246638		**HM143855**	Flower, Oeiras, Portugal

Species	Strains	D1/D2 LSU (GenBank)	ITS region (GenBank)	Mating behaviour Old	Mating behaviour New	*STE3 A1*	*STE3 A2*	*STE3.A1* (GenBank)	*STE3.A2* (GenBank)	*HD1/HD2* (GenBank)	Isolation source and location
R. babjevae	**PYCC 4915**	AF389825	AF387770	As		●		JN246639		**HM143853**	Pond water, Silwood, UK
R. babjevae	VKM Y-1310			A1		●		JN246640		JN246617	Silage, Kiev region, Ukraine
R. babjevae	VKM Y-1629			A2			●			**JN246618**	Soil, Krasnodar region, Russia
R. babjevae	VKM Y-1631			A2			●				Soil, Krasnodar region, Russia
R. babjevae	VKM Y-2911			Sf		●	●	JN246637	JN246574		Soil, Komi Republic, Russia
Rh. graminis	AJ 130			As			●		JN246578	**JN246634**	Soil in *Nothofagus* forest, Tongario National Park, New Zealand
Rh. graminis	AJ 132			As			●			**JN246635**	Soil in *Podocarpus* forest, Pureora Forest Park, New Zealand
Rh. graminis	**CBS 2826^{T}**	AF070431	AF444505	As			●		JN246576	**JN246632**	Grass, New Zealand
Rh. graminis	**CBS 3043**	AF387130	AF387780	As			●		JN246577	**JN246633**	Leaf of *Citrus* sp., Indonesia
Rh. graminis	WP1			n.d.			●		JGI Genome	**JGI Genome**	Isolated as an endophyte within *Populus trichocarpa* (black cottonwood), Western Washington state, USA
Rh. glutinis	**C13A1**	JN246532	JN246543	As		●		JN246643		**JN246631**	Fruiting body of *Cyttaria hariotii,* Patagonia, Argentina
Rh. glutinis	**CBS 20^{T}**	AF070430	AF387775	As		●		JN246644		**JN246626**	Air, unknown location
Rh. glutinis	CBS 2367			As		●		JN246647		**JN246627**	Fruit of *Phyllodendron* sp., Botanic Garden, Florence, Italy
Rh. glutinis	**JCM 3907**	DQ531948	AB073237	As		●		JN246642		JN246628	Unknown
Rh. glutinis	PYCC 4107			As		●		JN246646		JN246629	Seawater, Florida, USA
Rh. glutinis	**PYCC 5599**	AF387132	AF387777	As		●		JN246645		JN246630	Core samples from stratigraphic drillings, Japan
R. diobovatum	CBS 324			A1		●		JN246649		JN246623	Unknown substrate, Portugal
R. diobovatum	**CBS 6084**	AF189914	AB073236	A2			●		JN246579	JN246624	Seawater, South-eastern barrier reef, Florida, USA
R. diobovatum	**CBS 6085^{T}**	AF070421	AF387782	A1		●		JN246648		JN246625	Seawater, South-eastern barrier reef, Florida, USA
R. diobovatum	PYCC 2920			A2			●		JN246580	JN246622	Seawater, Scripps pier, USA
"Rh. pinicorticis"	**PYCC 4826^{T}**	AF387134	JN246544	As		○	○				Bark of *Pinus pinaster*, Sesimbra, Portugal
R. kratochvilovae	A113			As			●				Under thermocline, Algarve, Portugal
R. kratochvilovae	CBS 7293			Sf		●	●				Unknown
R. kratochvilovae	**CBS 7436^{T}**	AF071436	AF444520	Sf		●	●	JN246651	JN246584		Unknown
R. kratochvilovae	CRUB 0014			As		●		JN246652			*Rubus idaeus*, Patagonia, Argentina
R. kratochvilovae	CRUB 0121			A2			●				Lake Toncek, Argentina

**Species**	**Strains**	**D1/D2 LSU** (GenBank)	**ITS region** (GenBank)	**Mating behaviour** Old	New	***STE3* *A1***	***STE3* *A2***	***STE3.A1*** (GenBank)	***STE3.A2*** (GenBank)	***HD1/HD2*** (GenBank)	**Isolation source and location**
R. kratochvilovae	CRUB 1035			A2			●		JN246583		Lake Escondido, Argentina
R. kratochvilovae	PYCC 4776			A2			●				Plant leaf, Oeiras, Portugal
R. kratochvilovae	PYCC 4785			A2			●		JN246585		Humus, Arrábida, Portugal
R. kratochvilovae	**PYCC 4818**	AF1899116	AF444586	A1		●		JN246653			Wood chips, Setúbal, Portugal
R. kratochvilovae	**PYCC 4819**	AF189917	AF444587	A2			●		JN246581		Moss, Oeiras, Portugal
R. kratochvilovae	PYCC 5068			A2			●				Soil, Azores, Portugal
R. kratochvilovae	PYCC 5085			A2			●		JN246582		Soil, Azores, Portugal
R. kratochvilovae	PYCC 5244			A1		●		JN246650			Culture contaminant, Oeiras, Portugal
Rh. araucariae	**CBS 6031**[T]	AF070427	AF444510	As			●		JN246586		Rotting bark of *Araucaria araucana* (monkey-puzzle tree) Llaima, Chile
Rh. araucariae	CBS 7797			As			●		JN246587		Unknown
Rh. araucariae	**PYCC 4820**	AF387135	JN246545	As		●	●	JN246654	JN246588		Plant litter, Oeiras, Portugal
Rh. araucariae	PYCC 4821			As			●				Humus, Arrábida Natural Park, Portugal
Rh. araucariae	ZP 280			As			●				Plant litter, Sintra, Portugal
"Rh. hamamotoiana"	**PYCC 4724**	AF387137	JN246547	As			●		JN246589		Soil, Alentejo, Portugal
"Rh. hamamotoiana"	**PYCC 4824**[T]	AF387138	JN246546	As		●		JN246656			Rotten wood, Gerês, Portugal
"Rh. sesimbrana"	**PYCC 4691**[T]	AF387144	JN246548	As		●		JN246657			Bark of *Pinus pinaster*, Sesimbra, Portugal
R. paludigenum	CBS 4477			A2[(a)]		●		JN246658			Sea-water, Biscayne Bay, Florida, USA
R. paludigenum	CBS 6565			A1[(a)]			●		JN246591		*Rhizophora mangle* (mangrove), swamp, Southern Florida, USA
R. paludigenum	**CBS 6566**[T]	AF363640	AF363640	A2			●		JN246590		*Juncus roemerianus* (black rush), marsh, Florida, USA
R. paludigenum	**CBS 6567**	AF070424	AF444493	A1		●		JN246659			*Rhizophora mangle* (mangrove), swamp, Southern Florida, USA

[(a)] The molecular mating type of strains CBS 4477 and CBS 6565 is not in agreement with the original mating type designation. However, when crossing CBS 6566[T] and CBS 4474 a positive result was obtained, indicating that the analysed strains are in fact of opposite mating types. A confirmation of this result is needed for the original strains deposited at the CBS culture collection.

R. toruloides	A405			A2			●		JN246596		Rio Tinto, Spain
R. toruloides	A421			A1		●					Rio Tinto, Spain
R. toruloides	**CBS 14**	AF207884	AB049028	A1		●		JN246660			Wood pulp from Coniferae, Sweden
R. toruloides	CBS 315			A1		●		JN246663			Tokyo, Japan (type strain of *Rhodotorula rubescens*)
R. toruloides	CBS 350			A1		●					Wood pulp from conifer, Sweden

Species	Strains	D1/D2 LSU (GenBank)	ITS region (GenBank)	Mating behaviour Old	Mating behaviour New	*STE3 A1*	*STE3 A2*	*STE3.A1* (GenBank)	*STE3.A2* (GenBank)	*HD1/HD2* (GenBank)	Isolation source and location
R. toruloides	CBS 5745			A2			●		JN246594		Soil, Nishiyama oil field, Japan
R. toruloides	**CBS 6016**^{T}	CBS seq.	CBS seq.	Sf		●	●	JN246662	JN246595		Unknown, [Hybrid]: cross of IFO 0559 x IFO 0880 (CBS 14 X CBS 349)
R. toruloides	PYCC 4350			A2			●				Unknown
R. toruloides	PYCC 4661			A2			●				Woodland soil, Alentejo, Portugal
R. toruloides	PYCC 4786			A2			●				Dry leaf, Jardim Botânico, Rio de Janeiro, Brasil
R. toruloides	PYCC 4943			A2			●				Pond of solar saltern, Alcochete, Portugal
R. toruloides	**PYCC 5081**	JN246533	JN246549	A2			●		JN246593		Soil, Azores, Portugal
R. toruloides	PYCC 5082			A1		●		JN246661			Soil, Azores, Portugal
R. toruloides	PYCC 5109			A2			●		JN246592		Salt farm, Alcochete, Portugal
R. toruloides	SDY 231			A1		●					Acidic mine water, S. Domingos, Portugal
Rh. dairenensis	**CBS 2202**	JN246535	JN246550	As		○					Soil, USA
Rh. dairenensis	**CBS 4406**^{LT}	AF070429	AF444501	As			●		JN246597		Air, unknown location
Rh. dairenensis	CBS 7294			As		○					Unknown
Rh. dairenensis	**PYCC 4784**	AY033551	AF444683	As			●		JN246598		Plant litter, Arrábida, Portugal
Rh. dairenensis	PYCC 4897			As			●				Flower of *Canna indica*, Azores, Portugal
Rh. dairenensis	PYCC 4944			As			●				Salt farm, Alcochete, Portugal
Rh. dairenensis	PYCC 5601			As		○					Core samples from stratigraphic drillings, Japan
Rh. mucilaginosa	**CBS 17**	AF189960	AF444503	As		○	○				Unknown
Rh. mucilaginosa	**CBS 316**^{T}	AF070432	AF444541	As		○	○				Unknown
Rh. mucilaginosa	CBS 325			As		○	○				Air, Tokyo, Japan
Rh. mucilaginosa	CBS 326			As		○	○				Air, unknown location
Rh. mucilaginosa	CBS 992			As		○	○				Nail of 12-year-old girl, Austria
Rh. mucilaginosa	CBS 1011			As		○	○				Ulcer of man, Madagascar
Rh. mucilaginosa	CBS 2377			As		○	○				Skin, scrapings from axillary region of man,
Rh. mucilaginosa	CBS 2404			As		○	○				Fermenting Kentucky tobacco, Salerno, Italy
Rh. mucilaginosa	CBS 5804			As		○	○				Larvae of *Drosophila pilimanae* (fruit fly), Hawaii, USA
Rh. mucilaginosa	CBS 5951			As		○	○				Gut of *Guara rubra* (red ibis bird), Paris Zoological Park, France

Species	Strains	D1/D2 LSU (GenBank)	ITS region (GenBank)	Mating behaviour Old	Mating behaviour New	*STE3 A1*	*STE3 A2*	*STE3.A1* (GenBank)	*STE3.A2* (GenBank)	*HD1/HD2* (GenBank)	Isolation source and location
Rh. pacifica	**SY-246**	AB193175	AB193175	A1		○	○				Sediments, deep-sea floor (900 m), Iheya Ridge, Pacific Ocean
Rh. pacifica	**SY-96**[T]	AB026006	AB026006	A2		○	○				Sediments, deep-sea floor (3700 m), Yap Trench, Pacific Ocean
R. sphaerocarpum	**CBS 5939**[T]	AF070425	AF444499	A2	A1	○	○				Seawater, Marguerite Bay, Antarctica
R. sphaerocarpum	CBS 5940			A1	A2	○	○				Seawater, Gerlache Straits near Anvers Island, Antarctica
R. sphaerocarpum	**CBS 5941**	AF189919	AB073259	Sf		○	○				Sea water, Gerlache Straits near Brabant Island, Antarctica
R. sphaerocarpum	CBS 6985			A2	A1	○	○				Salt farm, Seto Inland Sea, Yoshima, Japan
R. sphaerocarpum	**PYCC 2988**	JN246534	JN246551	A2[(b)]	A1	🟠		JN246664			Unknown
R. sphaerocarpum	PYCC 5105			A2	A1	○	○				Salt farm, Alcochete, Portugal
R. sphaerocarpum	PYCC 5106			A2	A1	○	○				Salt farm, Alcochete, Portugal
R. sphaerocarpum	PYCC 5107			A1	A2	○	○				Salt farm, Alcochete, Portugal
R. sphaerocarpum	PYCC 5108			A1	A2	○	○				Salt farm, Alcochete, Portugal
R. sphaerocarpum	ZP 417			A2	A1	○	○				Mud from salt marsh, Tróia, Portugal
[(b)] *The molecular mating type of strain PYCC 2988 indicates that, despite originally designated as MAT A2, in fact is a MAT A1 strain. However, no positive results were obtained for the other strains of the same mating type.*											
Rh. taiwanensis	BCRC 23118[T]	GU646863	GUG46862			n.d.	n.d.				
"Rh. pyritica"	PYCC 4782			As		○	○				Wood of *Quercus suber*, Arrábida, Portugal
"Rh. pyritica"	**PYCC 5597**	AF387139	JN246552	As		○	○				Unknown (IAM 12959)
"Rh. pyritica"	RT 4.5.3			As		○	○				Acidic water, Rio Tinto, Spain
"Rh. pyritica"	**SDY 119**[T]	AY731799	JN246553	As		○	○				Acid mine water, S. Domingos, Portugal
"R. feracious"	**PYCC 5380**	AF387143	JN246554	As		○	○				Soil, Azores, Portugal
"R. feracious"	**SDY 020**[T]	AY731800	JN246555	Sf		○	○				Acidic mine water, S. Domingos, Portugal
"R. feracious"	SDY 080			As		○	○				Acidic mine water, S. Domingos, Portugal
"R. feracious"	SDY 176			Sf		○	○				Acidic mine water, S. Domingos, Portugal
R. azoricum	**CBS 4648**	DQ531944	JN246556	A1	A2		🔵		JN246600		Cacao, unknown location
R. azoricum	**CBS 8948**	AF321978	CBS Seq.	A2	A1	🟠		JN246655			Soil near thermal hot spring, São Miguel island, Azores, Portugal
R. azoricum	**CBS 8949**[T]	AF321977	CBS Seq.	A1	A2		🔵		JN246599		Soil, São Miguel island, Azores, Portugal
R. fluviale	**CBS 6568**[T]	AF189915	AY015432	Sf			🔵		JN246601		Brackish water, near mouth of Miami river, Florida, USA

Species	Strains	D1/D2 LSU (GenBank)	ITS region (GenBank)	Mating behaviour Old	Mating behaviour New	*STE3 A1*	*STE3 A2*	*STE3.A1* (GenBank)	*STE3.A2* (GenBank)	*HD1/HD2* (GenBank)	Isolation source and location
S. microsporus	**CBS 7041**^{T}			Sf			●		JN246602		Herbaceous culm, Jamaica
S. ruineniae	**CBS 5004**	AF387127	JN246558	A1	A2	○	○				Foliage of cacao tree (*Theobroma cacao*), Bogor, Indonesia
S. ruineniae	CBS 5078			As		○	○				Foliage of cacao tree (*Theobroma cacao*), Bogor, Indonesia
S. ruineniae	**CBS 5811**	AF070434	AB030339	As		○	○				Dung of goat, Pakistan
S. ruineniae	**CBS 9111**	AF387128	JN246557	A2	A1	●		JN246665			Garden soil, Taiwan
Sp. odoratus	**CBS 9115**	AF387125	CBS seq.	As		○	○				Basidiocarp of *Myxarium nucleatum*, Sesimbra, Portugal
Sp. odoratus	**ZP 470**	JN246540	JN246559	As		○	○				Culture contaminant, Caparica, Portugal
Sp. poonsookiae	**CBS 9095**^{T}	AF387124	AB030327	As		n.d.	n.d.				Dead leaf of *Mangifera indica*, Bangkok, Thailand
Sp. nylandii	**CBS 9093**^{T}	AF387123	AB030323	As		n.d.	n.d.				Dead leaf of *Oryza sativa*, Bangkok, Thailand
"*R. oreadum*"	**ZP 292**^{T}	JN246537	JN246560	A1		●		JN246666			Soil under snow, Serra da Estrela, Portugal
"*R. oreadum*"	**ZP 295**	JN246538	JN246561	A2		○	○				Soil under snow, Serra da Estrela, Portugal
Rh. colostri	A326			As			●		JN246605		Olo river, Portugal
Rh. colostri	C18B1			As			●				Fruiting body of *Cyttaria hariotii,* Patagonia, Argentina
Rh. colostri	**C21C1**	JN246536	JN246562	As		●		JN246667			Fruiting body of *Cyttaria hariotii,* Patagonia, Argentina
Rh. colostri	**CBS 348**^{T}	AY372177	JN246563	As			●		JN246603		Colostrum from woman
Rh. colostri	CBS 2201			As			●		JN246604		Unknown
Rh. colostri	CBS 9002			As		●		JN246668			Flowering plant of *Helleborus foetidus*, Marburg, Germany
Rh. colostri	ZP 970			As			●				Leaves of *Nothofagus mooreii*, Lamington National Park, Australia
Rh. colostri	ZP 984			As			●				*Cyttaria gunnii* on *Nothofagus cunninghamii*, Mt. Field Park, Tasmania
Rh. colostri	ZP 985			As			●				*Cyttaria gunnii* on *Nothofagus menziesii*, Lake Daniels, New Zealand
Rh. colostri	ZP 986			As			●				*Cyttaria gunnii* on *Nothofagus menziesii* (floor), Hasst Pass, New Zealand
R. lusitaniae	**CBS 7604**^{T}	AF070423	AY015430	Sf			●		JN246606		Soil of woodland, Lisbon, Portugal
R. lusitaniae	PYCC 4599			Sf			●		JN246607		Dried leaf material, Arrábida Natural Park, Portugal
R. lusitaniae	**PYCC 4642**	JN246539	AB087497	Sf			●		JN246608		Dried leaf material, Arrábida Natural Park, Portugal
Sp. ruberrimus	**CBS 7500**^{AUT}	AF070442	AY070004	As			●		JN246609		Air, Fukuoka, Japan

**Species**	**Strains**	**D1/D2 LSU** (GenBank)	**ITS region** (GenBank)	**Mating behaviour** Old	**Mating behaviour** New	***STE3 A1***	***STE3 A2***	***STE3.A1*** (GenBank)	***STE3.A2*** (GenBank)	***HD1/HD2*** (GenBank)	**Isolation source and location**
Sp. ruberrimus	**CRUB 1041**	JN246541	JN246564	As		🟡		JN246669			Aquatic environment, Patagonia, Argentina
Sp. phaffii	**CBS 9129**[T]	AY070011	AY069995	As		n.d.	n.d.				Wilting leaf of *Nerium indicum*, Yunnan, China
Sp. phaffii	**CH 2.049**	AY070010	AY069994	As		n.d.	n.d.				Wilting leaf of *Ehretia corylifolia*, Yunnan, China
Sporobolomyces sp.	**IAM 13481**	JGI Genome	JGI Genome	n.d.		🟡		JGI Genome			Leaf of willow, Japan (identified as *Sporobolomyces roseus*)
S. pararoseus	**CBS 484**	AF070437	AF417115	A1	A2		🔵		JN246610		Air, USA
S. pararoseus	**CBS 491**[T]	AF189977	AY015429	A2	A1	🟡		JN246670			Soil, Japan
S. pararoseus	CBS 499			A1	A2		🔵		JN246611		Air in dairy, USA
S. pararoseus	**CBS 7716**	EU003452	JN246565	A2	A1	🟡		JN246671			Soil, USSR
Sp. patagonicus	**CBS 9657**[T]	AY158655	AY552328	As		n.d.	n.d.				Subsurface water, lake Fonck, Patagonia, Argentina
Sp. patagonicus	**CBS 9658**	AY158656	AY552329	As		n.d.	n.d.				Subsurface water, lake Hess, Patagonia, Argentina
Sp. salmoneus	**CBS 488**[T]	AY070017	AY070005	As		n.d.	n.d.				Etiolated grass, under a wooden plank, Delft, The Netherlands
Sp. salmoneus	**CBS 4217**[T]	AF070440	AY069993	As		n.d.	n.d.				Air, unknown location
Sp. carnicolor	**CBS 4215**[AUT]	AY158641	AY069991	As		n.d.	n.d.				Unknown
Sp. japonicus	**CBS 5744**[T]	AY070009	AY069992	As		n.d.	n.d.				Oil brine, Yabase oil field, Akita Prefecture, Japan
Sp. bannaensis	**CBS 9204**[T]	AY274823	AY274824	As		n.d.	n.d.				Leaf of *Theobroma cacao*, Xishuang Banna, Yunnan, China
Sp. beijingensis	**CBS 9730**[T]	AY364837		As		n.d.	n.d.				Wilting leaf of *Sorbus pohuashanensis*, Baihua Mountain, Beijing, China
S. longiusculus	**CBS 9654**[T]	AY158657	AY552327	A1	A2		🔵		JN246612		Subsurface water, Fonck lake, Patagonia, Argentina
S. longiusculus	**CBS 9655**	AY552326	JN246566	A2	A1	○					Subsurface water, Ilon lake, Patagonia, Argentina
S. longiusculus	CBS 9656			A2	A1	○					Subsurface water, Ilon lake, Patagonia, Argentina
Sp. blumeae	**CBS 9094**[T]	AY070007	AB030331	As		🟡		JN246672			Dead leaf of *Blumea* sp., Bangkok, Thailand
S. metaroseus	**CBS 5541**	EU003458	EU003480	Sf		🟡		JN246673			Flower of *Fumaria* sp., France
S. metaroseus	**CBS 7683**[T]	EU003461	EU003482	Sf		○	○				Leaves, Portugal
Sp. jilinensis	**CBS 9728**[T]	AY364838		As		🟡		JN246674			Wilting leaf of *Pinus koraiensis*, Changbai Mountain, Jilin province, China
S. johnsonii	CBS 1522			As	As	🟡				GU474690	Fodder yeast, Germany
S. johnsonii	CBS 2630			A2	A1	🟡					Air, The Netherlands
S. johnsonii	**CBS 2634**	JN246542	JN246567	A1	A2		🔵		GU474647	**GU474685**	*Fragaria sp.*, Japan

Species	Strains	D1/D2 LSU (GenBank)	ITS region (GenBank)	Mating behaviour		*STE3 A1*	*STE3 A2*	*STE3.A1* (GenBank)	*STE3.A2* (GenBank)	*HD1/HD2* (GenBank)	Isolation source and location
				Old	New						
S. johnsonii	CBS 2643			A2	A1	●					Unknown substrate, Germany
S. johnsonii	CBS 4209			A2	A1	●					Fruit body of *Exidia* sp., Japan
S. johnsonii	**CBS 5470[T]**	AF070435	AY015431	Sf		**●**	**●**	GU474645	GU474648	**GU474692**	Leaf of *Rubus idaeus* with dead pustule of *Phragmidium rubi-idaei*, USA
S. johnsonii	**CBS 7795**	EF592139	EF592112	As		**●**		HM133777			Culture contaminant, Russia
S. johnsonii	CBS 8241			Sf		●	●				Unknown
S. johnsonii	PYCC 4351			A2	A1	**●**		GU474644		**GU474689**	Leaf of a tree, Portugal
S. salmonicolor	**CBS 483**	EF592144	EF592118	A2	A1	**●**		GU474642		**GU474673**	Rusted leaf of *Citrus* sp., France
S. salmonicolor	CBS 487			A1	A2		●				Air, Japan
S. salmonicolor	**CBS 490[T]**	AF070439	AY015434	A1	A2		**●**		GU474641	**GU474656**	Culture contaminant
S. salmonicolor	CBS 495			A1	A2		●				Culture contaminant, Japan
S. salmonicolor	CBS 496			A1	A2		●			GU474658	Scorched skin of orange, Delft, Netherlands
S. salmonicolor	CBS 497			A2	A1	●					Unknown
S. salmonicolor	CBS 1012			A1	A2		**●**		HM133782	**GU474649**	Leaf of *Aristolochia* sp., Botanic Laboratory, Delft, The Netherlands
S. salmonicolor	CBS 1013			A1	A2		●			**GU474650**	Air in dairy, USA
S. salmonicolor	CBS 1039			A1	A2		●			GU474651	Air in dairy, USA
S. salmonicolor	CBS 2635			A1	A2		●				Infected skin of a man, Germany
S. salmonicolor	CBS 2641			As			●				Air, Netherlands
S. salmonicolor	CBS 2647			A2	A1	●				GU474670	Gut of *Drosophila* sp., locality unknown
S. salmonicolor	CBS 2648			A2	A1	●					Exudate of *Kalopanax vicinifolium* var. *typicum*, Japan
S. salmonicolor	CBS 2873			A1	A2		●				Extract of oak bark, France
S. salmonicolor	CBS 4029			As			●				Soil, New Zealand
S. salmonicolor	CBS 4030			A1	A2		●				Soil, New Zealand
S. salmonicolor	CBS 4474			A2	A1	**●**		HM133770		**GU474672**	Blistered skin of a man, Bonn, Germany
S. salmonicolor	CBS 5937			A1	A2		●				Carious dentine, South Africa
S. salmonicolor	CBS 6322			A1	A2		**●**		HM133783	**GU474660**	Rotting wood shavings, Chile
S. salmonicolor	CBS 6470			A1	A2		●				Air, Germany
S. salmonicolor	CBS 6529			A2	A1	●					Culture contaminant, R.J. Bandoni
S. salmonicolor	CBS 6530			A1	A2		●				Bog, locality unknown

Species	Strains	D1/D2 LSU (GenBank)	ITS region (GenBank)	Mating behaviour Old	Mating behaviour New	*STE3 A1*	*STE3 A2*	*STE3.A1* (GenBank)	*STE3.A2* (GenBank)	*HD1/HD2* (GenBank)	Isolation source and location
S. salmonicolor	CBS 6781			A1	A2		●				Bituminous soil, oil field, Cumanà, Venezuela
S. salmonicolor	CBS 6832			A1	A2		**●**		GU474646	**GU474664**	Cerebro-spinal fluid, India
S. salmonicolor	CBS 7260			A1	A2		●			**GU474665**	Slime flux of *Quercus pendunculata*, Russia
S. salmonicolor	IHMT 2446/96			A2	A1	●				**GU474676**	Skin lesion, Portugal
S. salmonicolor	ML 2241			A2	A1	**●**		GU474643		**GU474678**	Isolated by J. W. Fell
S. salmonicolor	NRRL Y-17498			A2	A1	●					Ice core, Greenland
S. salmonicolor	PYCC 4558			A2	A1	**●**		HM133774		**GU474679**	Polluted river water, Oeiras, Portugal
S. salmonicolor	PYCC 4623			A2	A1	●				GU474680	Soil, USSR
S. salmonicolor	PYCC 5245			A1	A2		●				Unknown substrate, Portugal
S. salmonicolor	RJB 948			A2	A1	●					Isolated by R. J. Bandoni
S. salmonicolor	RJB 950			A1	A2		●				Isolated by R. J. Bandoni
S. salmonicolor	RJB 8219			A1	A2		●				Isolated by R. J. Bandoni
S. salmonicolor	ZP 392			A1	A2		●				Fruit body of *Dacrymyces* sp., Germany
S. salmonicolor	ZP 648			A2	A1	●				**GU474682**	Leaf of *Ligustrum* sp., Monte de Caparica, Portugal
