# Supplementary figures and images for: Evidence for maintenance of sex determinants but not of sexual stages in red yeasts, a group of early diverged basidiomycetes
Source: BMC Evol Biol. 2011 Aug 31;11:249. doi: 10.1186/1471-2148-11-249 (PMC3236058; doi:10.1186/1471-2148-11-249)

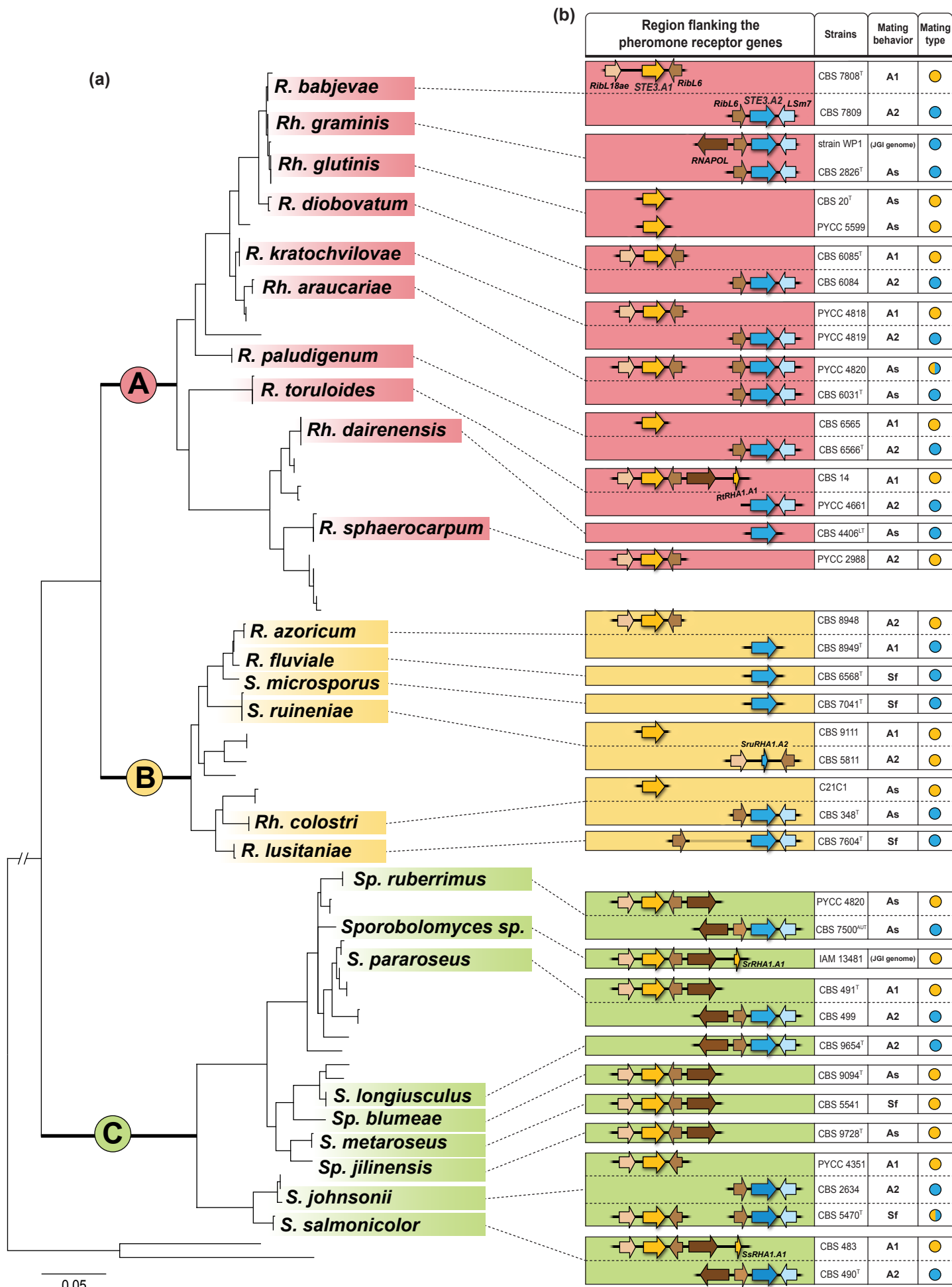

Supplement: Additional file 2 — Synteny of the genomic regions flanking the alternate PR in MAT A1 and MAT A2 strains of several red yeasts species. (a) Simplified illustration of the tree represented in Figure 1, indicating the phylogenetic placement of the species where gene organization in the vicinity of the pheromone receptor genes was determined. Clades A, B and C are the same as in Figure 1 and 2. (b) The mating behaviour (A1, A2, As and Sf stands for mating type A1, A2, asexual and self-fertile, respectively), the molecular mating type (STE3.A1, yellow circles; STE3.A2, dark blue circles; STE3.A1 and STE3.A2, half-coloured circles) and the obtained genomic regions flanking the pheromone receptor alleles (STE3.A1 and STE3.A2) are shown for each strain. Orthologues are shown in the same colour. In Rhodosporidium lusitaniae, the intervening region between the STE3.A2 and the RibL6 genes (faint line) was not sequenced. Abbreviations of generic names are as in Figure 1 and the remaining features are represented as in Figure 3. [file 1471-2148-11-249-S2.PDF]
