# Supplementary material for: Evidence for maintenance of sex determinants but not of sexual stages in red yeasts, a group of early diverged basidiomycetes
Source: BMC Evol Biol. 2011 Aug 31;11:249. doi: 10.1186/1471-2148-11-249 (PMC3236058; doi:10.1186/1471-2148-11-249)
Supplement: Additional file 3 — Pheromone precursors of different red yeast species. (a) The organization of the genomic region encompassing the MAT A1 pheromone precursor genes in Sporobolomyces sp. IAM 13481 is shown on top. Coding regions of the pheromone precursor genes (RHA1, RHA2 and RHA3) are depicted by gray arrows, indicating the direction of transcription. (b) Alignment of the Rha2 pheromone precursor of different MAT A1 red yeast species/strains (Ss, Sporidiobolus salmonicolor; Sj, Sporidiobolus johnsonii; Sp, Sporobolomyces sp. IAM 13481; Rt, Rhodosporidium toruloides). Amino acids differing from the S. salmonicolor strain CBS 483 are shown in red. Sequence repeats proposed to represent the peptide moiety of the mature pheromone are shadowed and those resembling the CAAX motif are underlined. (c) Phylogenetic tree showing the relationships between RHA2 genes from the indicated red yeast species, based on the alignment of their coding sequences. Groups are the same as in (b). Sequences of strains depicted in boldface are shown in (b). The tree was inferred using Maximum Parsimony. Bootstrap values from 1000 replicates are shown in the tree nodes. [file 1471-2148-11-249-S3.PDF]

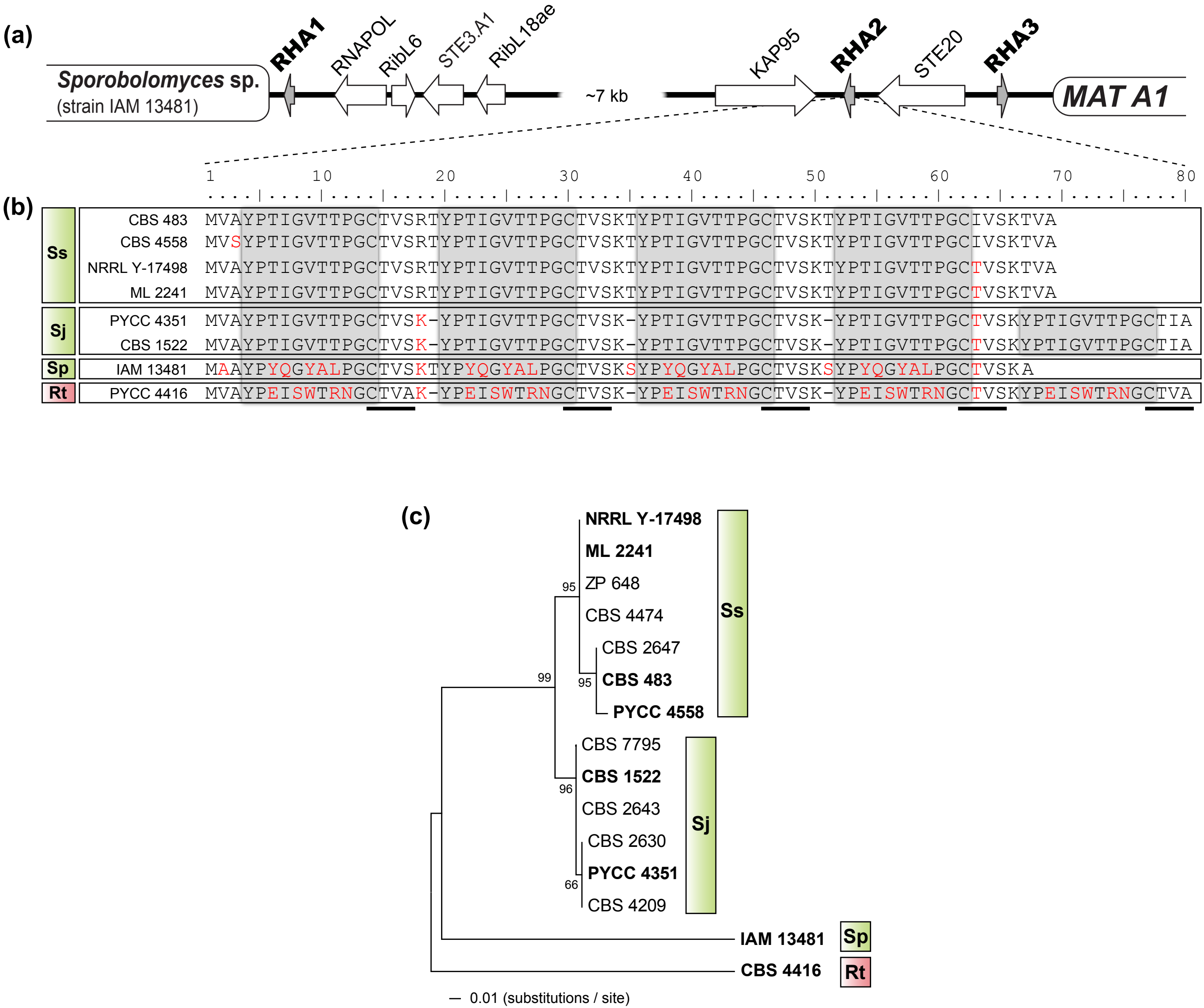

(a)
Sporobolomyces sp.
(strain IAM 13481)
RHA1
RNAPOL
RibL6
STE3.A1
RibL18ae
~7 kb
KAP95
RHA2
STE20
RHA3
MAT A1
(b)
1 10 20 30 40 50 60 70 80
Ss
CBS 483 MVAYPTIGVTTPGCTVSRTYPTIGVTTPGCTVSKTYPTIGVTTPGCTVSKTYPTIGVTTPGCIVSKTVA
CBS 4558 MVSYPTIGVTTPGCTVSRTYPTIGVTTPGCTVSKTYPTIGVTTPGCTVSKTYPTIGVTTPGCIVSKTVA
NRRL Y-17498 MVAYPTIGVTTPGCTVSRTYPTIGVTTPGCTVSKTYPTIGVTTPGCTVSKTYPTIGVTTPGCTVSKTVA
ML 2241 MVAYPTIGVTTPGCTVSRTYPTIGVTTPGCTVSKTYPTIGVTTPGCTVSKTYPTIGVTTPGCTVSKTVA
Sj
PYCC 4351 MVAYPTIGVTTPGCTVSK-YPTIGVTTPGCTVSK-YPTIGVTTPGCTVSK-YPTIGVTTPGCTVSKYPTIGVTTPGCTIA
CBS 1522 MVAYPTIGVTTPGCTVSK-YPTIGVTTPGCTVSK-YPTIGVTTPGCTVSK-YPTIGVTTPGCTVSKYPTIGVTTPGCTIA
Sp
IAM 13481 MAAYPYQGYALPGCTVSKTYPYQGYALPGCTVSKSYPYQGYALPGCTVSKSYPYQGYALPGCTVSKA
Rt
PYCC 4416 MVAYPEISWTRNGCTVAK-YPEISWTRNGCTVSK-YPEISWTRNGCTVSK-YPEISWTRNGCTVSKYPEISWTRNGCTVA
(c)
NRRL Y-17498
ML 2241
ZP 648
CBS 4474
CBS 2647
CBS 483
PYCC 4558
Ss
95
99
95
CBS 7795
CBS 1522
CBS 2643
CBS 2630
PYCC 4351
CBS 4209
Sj
96
66
IAM 13481
Sp
CBS 4416
Rt
0.01 (substitutions / site)
