# Supplementary material for: Evidence for maintenance of sex determinants but not of sexual stages in red yeasts, a group of early diverged basidiomycetes
Source: BMC Evol Biol. 2011 Aug 31;11:249. doi: 10.1186/1471-2148-11-249 (PMC3236058; doi:10.1186/1471-2148-11-249)
Supplement: Additional file 4 — Supplementary Table S1. Likelihood ratio statistics and parameter estimates for the dataset of clade A (S. salmonicolor and S. johnsonii) as inferred under seven models of ω over codons. Supplementary Table S2. Likelihood ratio statistics and parameter estimates for the dataset of clade B (R. babjevae, Rh. glutinis and Rh. graminis) as inferred under seven models of ω over codons. Supplementary Methods. Model characteristics and parameters for CODEML ctl file. [file 1471-2148-11-249-S4.PDF]

Additional File 4

Supplementary Table S1. Likelihood ratio statistics and parameter estimates for the dataset of clade A (*S. salmonicolor* and *S. johnsonii*) as inferred under seven models of ω over codons.

**Model** (no. parameters)	**LRT test**	**2Δ*l***	**df**		***p* value**	***l***	**ω** (d_N/d_S)	**Parameter estimate(s)**	**Positively selected sites** (BEB) (pp > 0.8)
M0 (one-ratio) (1)	M3 vs. M0	220.78	4	S	P << 0.001 (rejects M0)	-3324.79	0.504	ω = 0.504	
M3 (discrete) (5)						-3214.40	0.728	p_0 = 0.324 p_1 = 0.411 p_2 = 0.265<br>ω_0 = 0.058 ω_1 = 0.528 ω_2 = 1.860	
M1a (nearly Neutral) (2)	M2a vs. M1a	16.97	2	S	P < 0.001 (rejects M1a)	-3227.47	0.570	p_0 = 0.487 p_1 = 0.513<br>ω_0 = 0.117 ω_1 = 1.000	
M2a (positive selection) (4)						-3218.98	0.804	p_0 = 0.460 p_1 = 0.401 p_2 = 0.140<br>ω_0 = 0.125 ω_1 = 1.000 ω_2 = 2.483	21Y **60V** **84V** **86D** 86D<br>87T 105A
M7 (beta) (2)	M7 vs. M8	25.30	2	S	P < 0.001 (rejects M7)	-3227.18	0.515	p = 0.362 q = 0.340	
M8 (beta&ω) (4)						**-3214.53**	0.727	p_0 = 0.778 p_1 = 0.222<br>p = 0.541 q = 0.891 ω_s = 1.972	17R 21Y **60V** 64A 65R<br>79A **84V** 85Q **86D** **87T**<br>89K 94Y 96L 105A
M8a (beta&ω = 1) (3)	M8 vs. M8a	17.73	1	S	P < 0.001 (rejects M8a)	-3223.39	0.526	p_0 = 0.573 p_1 = 0.427<br>p = 0.790 q = 3.527 ω_s = 1.000	

Note: Positive selection sites are identified at the cutoff pp > 80%, with those with pp > 95% shown in **boldface**. "S" and "NS" stands for "significant" and "nonsignificant", respectively. All nested comparisons detected positively selected sites. M8 (beta&ω = 1) is the model that better describe this dataset (lower *l* values and less parameters). The 11 ω ratios under model M8 are 0.0045, 0.0343, 0.0880, 0.1630, 0.2576, 0.3696, 0.4970, 0.6371, 0.7858, 0.9350 and ω_s = 1.9724. The first 10 categories are from the β distribution, each with proportion 0.0778, and the last category has proportion 0.2220.

Supplementary Table S2. Likelihood ratio statistics and parameter estimates for the dataset of clade B (*R. babjevae*, *Rh. glutinis* and *Rh. graminis*) as inferred under seven models of ω over codons.

**Model** (no. parameters)	**LRT test**	**2Δ*l***	**df**	***p* value**		***l***	**ω** (d_N/d_S)	**Parameter estimate(s)**	**Positively selected sites** (BEB) (pp > 0.8)
M0 (one-ratio) (1)	M3 vs. M0	967.71	4	S	$P \ll 0.001$ (rejects M0)	-7485.50	0.172	ω = 0.172	
M3 (discrete) (5)						-7001.641	0.296	p_0 = 0.360 p_1 = 0.246 p_2 = 0.394<br>ω_0 = 0.018 ω_1 = 0.179 ω_2 = 0.623	
M1a (nearly Neutral) (2)	M2a vs. M1a	5.18	2	NS	$P > 0.05$	-7085.00	0.545	p_0 = 0.485 p_1 = 0.515<br>ω_0 = 0.061 ω_1 = 1.000	
M2a (positive selection) (4)						-7082.41	0.613	p_0 = 0.479 p_1 = 0.493 p_2 = 0.028<br>ω_0 = 0.062 ω_1 = 1.000 ω_2 = 3.204	
M7 (beta) (2)	M7 vs. M8	0.44	2	NS	$P > 0.05$	-6993.97	0.317	p = 0.386 q = 0.828	
M8 (beta&ω) (4)						-6993.75	0.330	p_0 = 1.000 p_1 = 0.000<br>p = 0.287 q = 1.065 ω_s = 2.511	
M8a (beta&ω = 1) (3)	M8 vs. M8a	-1.63	1			**-6992.93**	0.526	p_0 = 0.883 p_1 = 0.117<br>p = 0.428 q = 1.436 ω_s = 1.000	

Note: “S” and “NS” stands for “significant” and “nonsignificant”, respectively. All nested comparisons failed to reject the null hypothesis of no positively selected sites. M8a (beta&ω = 1) is the model that better describe this dataset providing some evidence that diversity is being generated due to relaxed purifying selection or relaxed functional constraints. The 11 ω ratios under model M8 are 0.0006, 0.0096, 0.0349, 0.0820, 0.1547, 0.2553, 0.3850, 0.5427, 0.7241, 0.9164, and ω_s = 1.8279. The first 10 categories are from the β distribution, each with proportion 0.0988, and the last category has proportion 0.0125.

Supplementary Methods

Model characteristics (parameters) based on references [1, 2]:

(i) **Model M0** (one-ratio) (1) assumes one ω (=d_N/d_S) for all codons in the sequence.

(ii) **Model M3** (discrete) (5) uses an unconstrained discrete distribution with three site classes estimated from the data.

(iii) **Model M1a** (nearly-neutral) (2) assumes two site classes estimated from the data, with $\omega_0 < 1$ and $\omega_1 = 1$.

(iv) **Model M2a** (positive selection) (4) adds a third class of sites to M1a, with $\omega_2 > 1$.

(v) **Model M7** (beta) (2) is a flexible null model, in which the ω ratio for a codon is a random draw from the β distribution with $0 < \omega < 1$.

(vi) **Model M8** (beta&ω) (4) adds an extra class of site to model M7, with a proportion of $\omega_s > 1$ estimated from the data.

(vii) **Model M8a** (beta&$\omega_s = 1$) (3) introduced by Swanson et al [2] its similar to model M8 except that the category ω_s is fixed at $\omega_s = 1$ (specified in CODEML using NSsites = 8, fix omega = 1 and omega = 1) and thus not allowing positively selected sites.

Model comparisons using LRTs (H_0 – null hypothesis):

(i) **M0** (H_0) vs. **M3** tests for variation of ω among codons within the analysed region, using 4 degrees of freedom (df).

(ii) **M1a** (H_0) vs. **M2a** and **M7** (H_0) vs. **M8** tests whether or not the analysed region evolve under positive selection [the two models that allow a class of codons with positively selected sites (i.e. $\omega > 1$ in models M2a and M8) are compared to their nested neutral models (M1 and M7, respectively), using 2 df] [3]

(iii) **M8a** (H_0) vs. **M8** test for evidence of positive selection while eliminating the potential identification of relaxed purifying selection.

CODEML ctl file (models M0 to M8)

```
      seqfile = *******.phylips  * sequence data filename
     treefile = *******.nwk  * tree structure file
      outfile = results.txt  * main result file name
        noisy = 3  * 0,1,2,3,9: how much rubbish on the screen
      verbose = 1  * 0: concise; 1: detailed, 2: too much
      runmode = 0  * 0: user tree;  1: semi-automatic;  2: automatic
                   * 3: StepwiseAddition; (4,5):PerturbationNNI; -2: pairwise
      seqtype = 1  * 1:codons; 2:AAs; 3:codons-->AAs
    CodonFreq = 2  * 0:1/61 each, 1:F1X4, 2:F3X4, 3:codon table
        model = 0  *
      NSsites = 0 1 2 3 7 8 *
        icode = 0  * 0:universal code; 1:mammalian mt; 2-10:see below
    fix_kappa = 0  * 1: kappa fixed, 0: kappa to be estimated
        kappa = 2  * initial or fixed kappa
    fix_omega = 0  * 1: omega or omega_1 fixed, 0: estimate
        omega = 5  * initial or fixed omega, for codons or codon-based AAs
```

CODEML ctl file (model M8a)

```
      seqfile = *******.phylips * sequence data filename
     treefile = *******.nwk
      outfile = results.txt * main result file name
        noisy = 3  * 0,1,2,3,9: how much rubbish on the screen
      verbose = 1  * 0: concise; 1: detailed, 2: too much
      runmode = 0  * 0: user tree;  1: semi-automatic;  2: automatic
                   * 3: StepwiseAddition; (4,5):PerturbationNNI; -2: pairwise
      seqtype = 1  * 1:codons; 2:AAs; 3:codons-->AAs
    CodonFreq = 2  * 0:1/61 each, 1:F1X4, 2:F3X4, 3:codon table
        model = 0  *
      NSsites = 8  *
        icode = 0  * 0:universal code; 1:mammalian mt; 2-10:see below
    fix_kappa = 0  * 1: kappa fixed, 0: kappa to be estimated
        kappa = 2  * initial or fixed kappa
    fix_omega = 1  * 1: omega or omega_1 fixed, 0: estimate
        omega = 1  * initial or fixed omega, for codons or codon-based AAs
```

References

1. Nielsen R, Yang Z: **Likelihood models for detecting positively selected amino acid sites and applications to the HIV-1 envelope gene.** *Genetics* 1998, **148:**929-936.

2. Swanson WJ, Nielsen R, Yang Q: **Pervasive adaptive evolution in mammalian fertilization proteins.** *Mol Biol Evol* 2003, **20:**18-20.

3. Yang Z, Nielsen R, Goldman N, Pedersen A-MK: **Codon-substitution models for heterogeneous selection pressure at amino acid sites.** *Genetics* 2000, **155:**431-449.
