# Supplementary material for: Evidence for maintenance of sex determinants but not of sexual stages in red yeasts, a group of early diverged basidiomycetes
Source: BMC Evol Biol. 2011 Aug 31;11:249. doi: 10.1186/1471-2148-11-249 (PMC3236058; doi:10.1186/1471-2148-11-249)

Additional File 5. List of primers and specific PCR conditions used to amplify the indicated regions in red yeast species. Two PCR protocols (**A** and **B**) shown below were used to amplify the different genomic regions under study. For each region, it is indicated which of two protocols was used. Primer annealing sites are shown at the end for each studied region.

A. For PCR screening, reactions were performed in a final volume of 10 μl with the following components: 1X DreamTaq Buffer (Fermentas, Canada), 0.20 mM of each of the four dNTPs (GE Healthcare), 1% DMSO, 1.0 μM of each primer, 100 ng of genomic DNA, and 0.5 U DreamTaq DNA polymerase (Fermentas, Canada). Thermal cycling consisted of a 5-minute denaturation step at 95°C, followed by 35 cycles of denaturation at 95°C for 30 s, 30 s at the annealing temperature (variable), and extension at 72°C (variable time). The annealing temperatures, extension times and primer sequences are given for each case. A final extension of 7 min at 72°C was performed at the end of each reaction. PCRs reactions were adjusted to 50 μl final volume when sequencing was required.

B. For the long range PCR (LR-PCR), reactions were performed in a final volume of 50 μl and contained the following components: 1X Long PCR buffer, 2 mM of $MgCl_2$, 0.2 mM of each of the four dNTPs (GE healthcare), 1.0% DMSO, 1.0 μM of primer, 150 ng of genomic DNA and 2.5 U Long PCR enzyme mix (Fermentas, Canada). Thermal cycling consisted of a 3-minute denaturation step at 94ºC, followed by an initial round of 10 cycles of denaturation at 95ºC for 20 s, annealing for 30 s (variable temperature), extension at 68ºC (variable time), and a second round of 25 cycles increasing the extension time in each cycle (variable). A final extension of 10 minutes at 68ºC was performed. The annealing temperatures, extension times and primer sequences are given for each case.

Additional File 5. Continued.

**Amplification of *STE3. A1* allele – Protocol A**			
**Species**	**Primer Name**	**Sequence (5' → 3')**	**Annealing/ Extension**
R. babjevae, *Rh. glutinis*, *R. diobovatum*, *Rh. araucariae*, *R. kratochvilovae*, *"Rh. hamamotoiana"*, *R. sphaerocarpum*, *R. azoricum*, *"R. oreadum"*, *Rh. colostri*,	**MC159**<br>**MC164**	ACGYTGCTSTTCATYTTYTGGTG<br>GAAGATSGCRCASGAGATKGCGTA	60.0ºC / 60 s
"Rh. sesimbrana", *R. paludigenum*, *S. ruineniae*	**MC190**<br>**MC191**	ACCYTGCTYCTCATYTTYTGG<br>ATGGCGCTYTTKGACGAYTC	59.0ºC / 60 s
R. toruloides	**MC057**<br>**MC058**	CATCTTCTGGTGTKTGGTCAMCGT<br>GGGASGSTGTAGGCGATTTG	60.0ºC / 60 s
S. salmonicolor, *S. johnsonii*	**MC053**<br>**MC054**	CATCTTCTTCCTCCTCCTGCC<br>GGGTAGGGAGGAAGCGAGAT	59.5ºC / 90 s
**Amplification of the intervening region between *RibL18ae* and *RNAPOL* genes (encompasses the *STE3. A1*) – Protocol B**			
**Species**	**Primer Name**	**Sequence (5' → 3')**	**Annealing/ Extension**
S. metaroseus , *S. pararoseus*	**MC135**<br>**MC138**	GAGAAGGAGCCKCARCCCAA<br>CCTGTCCKCCKCGCATCTT	60ºC / 7 min – 10 cy.<br>60ºC / 7 min + 5 s/ cy. – 25 cy.
	**MC188**<br>**MC189**	ACCGWCCMTCGACYTTCTACT<br>ACTCACRCTCTCVACACGCTC	60ºC / 4 min – 10 cy.<br>60ºC / 4 min + 2 s/ cy. – 25 cy.
	**MC160**<br>**MC192**	ACTYTCMTCTACATYTTYTGGTG<br>CACCCTCACYTATATTTTCTGG	Primer-walking sequencing (*S. metaroseus*)<br>Primer-walking sequencing (*S. pararoseus*)
Sp. ruberrimus, *Sp. blumeae*	**MC188**<br>**MC189**	ACCGWCCMTCGACYTTCTACT<br>ACTCACRCTCTCVACACGCTC	60ºC / 4 min – 10 cy.<br>60ºC / 4 min + 2 s/ cy. – 25 cy.
	**MC160**	ACTYTCMTCTACATYTTYTGGTG	Primer-walking sequencing
Sp. jilinensis	**MC188**<br>**MC189**	ACCGWCCMTCGACYTTCTACT<br>ACTCACRCTCTCVACACGCTC	60ºC / 4 min – 10 cy.<br>60ºC / 4 min + 2 s/ cy. – 25 cy.
	**MC193**	CACCTTGATCTACGTTTTCTGG	Primer-walking sequencing
**No amplification of the *STE3.A1* allele was obtained in the following species using any combination of the above mentioned primers**			
Rh. graminis, *"Rh. pinicortis"*, *R. dairenensis, Rh. mucilaginosa, Rh. pacifica, "Rh. pyritica", "R. feracious"*, *R. fluviale*, *S. microsporus*, *Sp. odoratus*, *R. lusitaniae*, *S. longiusculus*			

Additional File 5. Continued.

Amplification of *STE3. A2* allele – Protocol A			
**Species**	**Primer Name**	**Sequence (5' → 3')**	**Annealing/ Extension**
R. babjevae, *Rh. graminis*, *R. kratochvilovae*,	**MC124**<br>**MC125**	AACAKCGSGCCWCTGTACTGGCAG<br>GCSACSACCCGAYSAAAGTTGCG	63.0ºC / 60 s
S. salmonicolor, *S. johnsonii*	**MC126**<br>**MC127**	AATRCCGCTCCGCTYTACTGGCA<br>TGTTCGGGTCTACAGGTTGGAGAG	62.0ºC / 90 s
R. diobovatum, *Rh. araucariae*, *"Rh. hamamotoiana"*, *R. paludigenum*, *R. toruloides*, *Rh. dairenensis*, *R. azoricum*, *R. fluviale*, *S. microsporus*, *R. lusitaniae*	**MC166**<br>**MC169**	AKCGTKCCKCTSTACTGGCA<br>CCGAAMGCGGCRAARAARAT	58.0ºC / 90 s
Rh. colostri	**MC166**<br>**MC168**	AKCGTKCCKCTSTACTGGCA<br>CYGAAMGCSGCGAAGAARAT	59.0ºC / 60 s
**Amplification of the intervening region between *LSm7* and *RNAPOL* genes (encompasses the *STE3. A2*) – Protocol B**			
Sp. ruberrimus	**MC123**<br>**MC189**	AAAGGSTACGACCAGYTSCTCAAYCT<br>ACTCACRCTCTCVACACGCTC	62ºC / 4 min – 10 cy.<br>62ºC / 4 min + 2 s/ cy. – 25 cy.
	**MC195**<br>**MC198**	TACAGGGTAARGGTATCTT<br>CTGACAGGACGCATAGATACAT	54ºC / 2 min – 10 cy.<br>54ºC / 2 min + 1 s/ cy. – 25 cy.
Sp. pararoseus	**MC123**<br>**MC189**	AAAGGSTACGACCAGYTSCTCAAYCT<br>ACTCACRCTCTCVACACGCTC	62ºC / 4 min – 10 cy.<br>62ºC / 4 min + 2 s/ cy. – 25 cy.
	**MC196**<br>**MC199**	GCGAAGTHGCWGCTCGAATC<br>CCGATAGCCGTCCAACAGG	60ºC / 2 min – 10 cy.<br>60ºC / 2 min + 1 s/ cy. – 25 cy.
S. longiusculus	**MC123**<br>**MC189**	AAAGGSTACGACCAGYTSCTCAAYCT<br>ACTCACRCTCTCVACACGCTC	62ºC / 4 min – 10 cy.<br>62ºC / 4 min + 2 s/ cy. – 25 cy.
	**MC194**<br>**MC197**	CCGTGAAGGACGCCTCGAT<br>CTCTCGCTCAATGACTACCTT	59ºC / 2 min – 10 cy.<br>59ºC / 2 min + 1 s/ cy. – 25 cy.
	**MC167**	AATRCCGCKCCKCTYTACTGGCA	Primer-walking sequencing
**No amplification of the *STE3.A2* allele was obtained in the following species using any combination of the abovementioned primers**			
Rh. glutinis, *"Rh. sesimbrana"*, *"Rh. pinicortis"*, *Rh. mucilaginosa, Rh. pacifica, R. sphaerocarpum*, *"Rh. pyritica", "R. feracious", Sp. odoratus*, *"R. oreadum"*, *S. longiusculus*, *Sp. blumeae*, *Sp. jilinensis*			

Additional File 5. Continued.

Confirm synteny in the vicinity of the *STE3.A1* allele in selected red yeast species (see Figure 3 and Additional File 2) **– Protocols A** and **B**

Amplicons were obtained for the following red species but only fully sequenced in those for which a GenBank Accession number is given

Species	Primer Name	Sequence (5' → 3')	Annealing/ Extension	Amplified region	GenBank Accession Number
R. babjevae, R. diobovatum	**MC109**<br>**MC122**	CGTCCGCCGCCCGAACATCAAG<br>TGGCGMGTYCCCGAACCYTACAAC	65ºC / 4 min – 10 cy.<br>65ºC / 4 min + 2 s/ cy. – 25 cy.	*Rib L18ae – RibL6*<br>(includes the *STE3.A1*)	
R. kratochvilovae					JN246653
Rh. araucariae					JN246654
R. azoricum					JN246655
R. toruloides	**MC109**<br>**MC161**	CGTCCGCCGCCCGAACATCAAG<br>AAGAYSGCCGGCAKGCTCCARTA	65ºC / 4 min – 10 cy.<br>65ºC / 4 min + 2 s/ cy. – 25 cy.	*Rib L18ae – RtSTE3.A1*	
	**MC057**<br>**MC022**	CATCTTCTGGTGTKTGGTCAMCGT<br>GGCGGATGGATTGGTTGAGC	60ºC / 6 min – 10 cy.<br>60ºC / 6 min + 2 s/ cy. – 25 cy.	*RtSTE3.A1 – RHA1*	[ref. 36]
R. sphaerocarpum	**MC109**<br>**MC122**	CGTCCGCCGCCCGAACATCAAG<br>TGGCGMGTYCCCGAACCYTACAAC	65ºC / 4 min – 10 cy.<br>65ºC / 4 min + 2 s/ cy. – 25 cy.	*Rib L18ae – RibL6*<br>(includes the *STE3.A1*)	JN246664
	**MC159**<br>**MC164**	ACGYTGCTSTTCATYTTYTGGTG<br>GAAGATSGCRCASGAGATKGCGTA	60.0ºC / 60 s		
Sp. ruberrimus, *Sp. blumeae*	**MC188**<br>**MC189**	ACCGWCCMTCGACYTTCTACT<br>ACTCACRCTCTCVACACGCTC	60ºC / 4 min – 10 cy.<br>60ºC / 4 min + 2 s/ cy. – 25 cy.	*Rib L18ae – RNAPOL*<br>(includes the *STE3.A1* and *RibL6* genes)	
	**MC160**	ACTYTCMTCTACATYTTYTGGTG	Primer-walking sequencing		
S. pararoseus	**MC135**<br>**MC138**	GAGAAGGAGCCKCARCCCAA<br>CCTGTCCKCCKCGCATCTT	60ºC / 4 min – 10 cy.<br>60ºC / 4 min + 2 s/ cy. – 25 cy.	*Rib L18ae – RNAPOL*<br>(includes the *STE3.A1* and *RibL6* genes)	JN246670
	**MC188**<br>**MC189**	ACCGWCCMTCGACYTTCTACT<br>ACTCACRCTCTCVACACGCTC	Primer-walking sequencing		
	**MC192**	CACCCTCACYTATATTTTCTGG			

Additional File 5. Continued.

Species	Primer Name	Sequence (5' → 3')	Annealing/ Extension	Amplified region	GenBank Accession Number
S. metaroseus	**MC135**<br>**MC138**	GAGAAGGAGCCKCARCCCAA<br>CCTGTCCKCCKCGCATCTT	60ºC / 4 min – 10 cy.<br>60ºC / 4 min + 2 s/ cy. – 25 cy.	*Rib L18ae – RNAPOL*<br>(includes the *STE3.A1* and *RibL6* genes)	
	**MC188**<br>**MC189**	ACCGWCCMTCGACYTTCTACT<br>ACTCACRCTCTCVACACGCTC	Primer-walking sequencing		
	**MC160**	ACTYTCMTCTACATYTTYTGGTG			
Sp. jilinensis	**MC188**<br>**MC189**	ACCGWCCMTCGACYTTCTACT<br>ACTCACRCTCTCVACACGCTC	60ºC / 4 min – 10 cy.<br>60ºC / 4 min + 2 s/ cy. – 25 cy.	*Rib L18ae – RNAPOL*<br>(includes the *STE3.A1* and *RibL6* genes)	
	**MC193**	CACCTTGATCTACGTTTTCTGG	Primer-walking sequencing		
S. ruineniae	**MC109**<br>**MC122**	CGTCCGCCGCCCGAACATCAAG<br>TGGCGMGTYCCCGAACCYTACAAC	65ºC / 4 min – 10 cy.<br>65ºC / 4 min + 2 s/ cy. – 25 cy.	*Rib L18ae – RibL6*<br>(includes the *SruRHA1.A2*)	

Confirm synteny in the vicinity of the *STE3.A2* allele in selected red yeast species (see Figure 3 and Additional File 2) **– Protocols A** and **B**

Amplicons were obtained for the following red species but only fully sequenced in those for which a GenBank Accession number is given

Species	Primer Name	Sequence (5' → 3')	Annealing/ Extension	Amplified region	GenBank Accession Number
R. babjevae, *Rh. graminis*, *R. lusitaniae*	**MC122**<br>**MC123**	TGGCGMGTYCCCGAACCYTACAAC<br>AAAGGSTACGACCAGYTSCTCAAYCT	(LR-PCR)<br>63ºC / 4 min – 10 cy.<br>63ºC / 4 min + 2 s/ cy. – 25 cy.	*LSm7 – RibL6*<br>(includes the *STE3.A2*)	
R. diobovatum					JN246579
R. kratochvilovae					JN246585
Rh. araucariae					JN246588
Rh. colostri					JN246603
S. johnsonii					JN246613
R. paludigenum, *R. toruloides*	**MC123**<br>**MC124**	AAAGGSTACGACCAGYTSCTCAAYCT<br>AACAKCGSGCCWCTGTACTGGCAG	62.0ºC / 2 min	*STE3.A2 – LSm7*	

Additional File 5. Continued.

Species	Primer Name	Sequence (5' → 3')	Annealing/ Extension	Amplified region	GenBank Accession Number
Sp. ruberrimus	**MC123**<br>**MC189**	AAAGGSTACGACCAGYTSCTCAAYCT<br>ACTCACRCTCTCVACACGCTC	62ºC / 4 min – 10 cy.<br>62ºC / 4 min + 2 s/ cy. – 25 cy.	*LSm7 – RNAPOL*<br><br>(includes the *STE3.A2 and RibL6* genes)	
	**MC195**<br>**MC198**	TACAGGGTAARGGTATCTT<br>CTGACAGGACGCATAGATACAT	54ºC / 2 min – 10 cy.<br>54ºC / 2 min + 1 s/ cy. – 25 cy.		
Sp. pararoseus	**MC123**<br>**MC189**	AAAGGSTACGACCAGYTSCTCAAYCT<br>ACTCACRCTCTCVACACGCTC	62ºC / 4 min – 10 cy.<br>62ºC / 4 min + 2 s/ cy. – 25 cy.	*LSm7 – RNAPOL*<br><br>(includes the *STE3.A2 and RibL6* genes)	JN246610<br>JN246611
	**MC196**<br>**MC199**	GCGAAGTHGCWGCTCGAATC<br>CCGATAGCCGTCCAACAGG	60ºC / 2 min – 10 cy.<br>60ºC / 2 min + 1 s/ cy. – 25 cy.		
S. longiusculus	**MC123**<br>**MC189**	AAAGGSTACGACCAGYTSCTCAAYCT<br>ACTCACRCTCTCVACACGCTC	(LR-PCR)<br>62ºC / 4 min – 10 cy.<br>62ºC / 4 min + 2 s/ cy. – 25 cy.	*LSm7 – RNAPOL*<br><br>(includes the *STE3.A2 and RibL6* genes)	JN246612
	**MC194**<br>**MC197**	CCGTGAAGGACGCCTCGAT<br>CTCTCGCTCAATGACTACCTT	(LR-PCR)<br>59ºC / 2 min – 10 cy.<br>59ºC / 2 min + 1 s/ cy. – 25 cy.		
	**MC167**	AATRCCGCKCCKCTYTACTGGCA	Primer-walking sequencing		
S. salmonicolor	**MC123**<br>**MC189**	AAAGGSTACGACCAGYTSCTCAAYCT<br>ACTCACRCTCTCVACACGCTC	62ºC / 4 min – 10 cy.<br>62ºC / 4 min + 2 s/ cy. – 25 cy.	*LSm7 – RNAPOL*<br><br>(includes the *STE3.A2 and RibL6* genes)	
	**MC126**<br>**MC122**	AATRCCGCTCCGCTYTACTGGCA<br>TGGCGMGTYCCCGAACCYTACAAC	62ºC / 2 min – 10 cy.<br>62ºC / 2 min + 1 s/ cy. – 25 cy.		

**Amplification of *HD1/HD2* alleles – Protocol B**			
**Species**	**Primer Name**	**Sequence (5' → 3')**	**Annealing/ Extension**
R. babjevae, *Rh. glutinis, Rh. graminis*, *R. diobovatum*,	**MC118**<br>**MC120**	TGTTGRYGAACCAGGTRTCGAKCTG<br>AGGATGKCGAGGACSTCGKGSGTGAA	65ºC / 1 min 30 s – 10 cy.<br>65ºC / 1 min 30 s + 1 s/ cy. – 25 cy.
S. salmonicolor, *S. johnsonii*	See Reference [21] of the manuscript		

Additional File 5. Continued.

Amplification intervening region between *STE20* and *KAP95* genes (encompasses the *RHA2*) – Protocol A					
**Species**	**Primer Name**	**Sequence (5' → 3')**	**Annealing/ Extension**	**Amplified region**	**GenBank Accession Number**
S. salmonicolor (ML 2241, NRRL Y-17498)<br>*S. johnsonii* (CBS 1522)	**MC040**<br>**MC073**	GCCCGAAGTCGTCAAGCAGAAGGA<br>TTCCCSAAYGGCCARCTCAAGGAGCC	63.0ºC / 3 min	*STE20 – KAP95*<br>(includes the *RHA2* genes)	JN246569<br>JN246568<br>JN246571
	**MC113**<br>**MC115**	AGCCRGCGCTTSGGATCAGTTAC<br>AAGGAAGGATGCTYACTTGCGG	Primer-walking sequencing		
S. salmonicolor (PYCC 4558)	**MC040**<br>**MC073**	GCCCGAAGTCGTCAAGCAGAAGGA<br>TTCCCSAAYGGCCARCTCAAGGAGCC	63.0ºC / 3 min	*STE20 – KAP95*<br>(includes the *RHA2* genes)	JN246570
	**MC113**<br>**MC115**<br>**MC130**	AGCCRGCGCTTSGGATCAGTTAC<br>AAGGAAGGATGCTYACTTGCGG<br>TCCGAACAATGGATCAACAGGC	Primer-walking sequencing		
S. jonhsonii (strains: PYCC 4351)	**MC040**<br>**MC073**	GCCCGAAGTCGTCAAGCAGAAGGA<br>TTCCCSAAYGGCCARCTCAAGGAGCC	63.0ºC / 3 min	*STE20 – KAP95*<br>(includes the *RHA2* genes)	JN246572
	**MC114**<br>**MC115**	GGAATCGGGACCCCAAGAAAC<br>AAGGAAGGATGCTYACTTGCGG	Primer-walking sequencing		
R. toruloides	See Reference [21] of the manuscript				

Additional File 5. Continued.

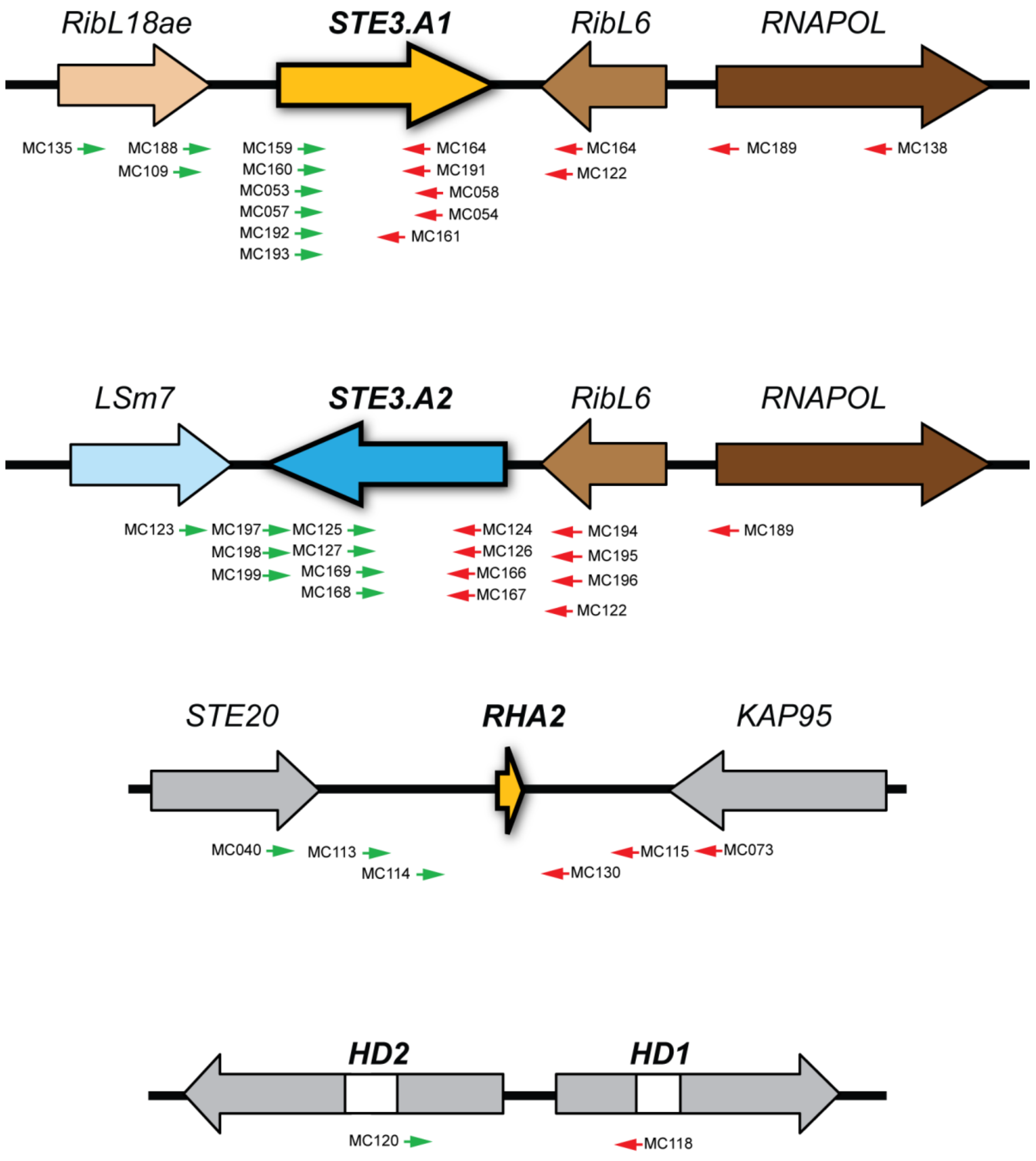

Supplement: Additional file 5 — List of primers and specific PCR conditions used to amplify the indicated regions. [file 1471-2148-11-249-S5.PDF]
